# Species, taxonomic, and functional group diversities of terrestrial mammals at risk under climate change and land‐use/cover change scenarios in Mexico

**DOI:** 10.1111/gcb.16411

**Published:** 2022-09-13

**Authors:** Carolina Ureta, Mercedes Ramírez‐Barrón, Edgar Andrés Sánchez‐García, Angela P. Cuervo‐Robayo, Mariana Munguía‐Carrara, Alma Mendoza‐Ponce, Carlos Gay, Víctor Sánchez‐Cordero

**Affiliations:** ^1^ Instituto de Ciencias de la Atmósfera y Cambio Climático, Universidad Nacional Autónoma de México Ciudad de México Mexico; ^2^ Investigadora por México‐CONACyT Consejo Nacional de Ciencia y Tecnología Ciudad de México Mexico; ^3^ Departamento de Zoología Instituto de Biología, Universidad Nacional Autónoma de México Ciudad de México Mexico; ^4^ Comisión Nacional para el Conocimiento y Uso de la Biodiversidad (CONABIO), Insurgentes Sur‐Periférico Ciudad de México Mexico; ^5^ International Institute for Applied Systems Analysis Laxenburg Austria

**Keywords:** climate change, exposure, hazard, IPCC, land‐use changes, species risk index, vulnerability

## Abstract

There is a need to revise the framework used to project species risks under climate change (CC) and land‐use/cover change (LUCC) scenarios. We built a CC risk index using the latest Intergovernmental Panel on Climate Change framework, where risk is a function of vulnerability (sensitivity and adaptive capacity), exposure, and hazard. We incorporated future LUCC scenarios as part of the exposure component. We combined a trait‐based approach based on biological characteristics of species with a correlative approach based on ecological niche modeling, assigning risk scores to species, taxonomic (orders), and functional (trophic, body size, and locomotion) groups of terrestrial mammals occurring in Mexico. We identified 15 species projected to lose their climatic suitability. Of the 11 taxonomic orders, Eulipotyphla, Didelphimorphia, Artiodactyla, and Lagomorpha had the highest risk scores. Of the 19 trophic groups, piscivores, insectivores under canopy, frugivores–granivores, herbivores browser, and myrmecophagous had the highest risk scores. Of the five body‐sized groups, large‐sized species (>15 kg) had highest risk scores. Of the seven locomotion groups, arboreal and semi‐aquatics had highest risk scores. CC and LUCC scenarios reduced suitable areas of species potential distributions by 37.5% (with CC), and 51% (with CC and LUCC) under a limited full‐dispersal assumption. Reductions in suitable areas of species potential distributions increased to 50.2% (with CC), and 52.4% (with CC and LUCC) under a non‐dispersal assumption. Species‐rich areas (>75% species) projected 36% (with CC) and 57% (with CC and LUCC) reductions in suitability for 2070. Shifts in climatic suitability projections of species‐rich areas increased in number of species in northeast and southeast Mexico and decreased in northwest and southern Mexico, suggesting important species turnover. High‐risk projections under future CC and LUCC scenarios for species, taxonomic, and functional group diversities, and species‐rich areas of terrestrial mammals highlight trends in different impacts on biodiversity and ecosystem function.

## Graphical abstract

We combined trait‐based and correlative approaches to assign risks to species, taxonomic orders, and functional groups of terrestrial mammals in Mexico, under climate change (CC) and land‐use/cover change (LUCC) scenarios. Risk scores were obtained as a function of vulnerability, exposure, and hazard, using the latest Intergovernmental Panel on Climate Change framework. Overall, species (20), taxonomic orders (Eulipotyphla, Didelphimorphia, Artiodactyla, and Lagomorpha), and functional groups (large‐sized species >15 kg, piscivores, insectivores‐under‐canopy, frugivores–granivores, herbivores–browser, myrmecophagous, arboreal, and semi‐aquatic species) showed highest risk. Species‐rich areas projected 36% (with CC) and 57% (with CC and LUCC) reductions in suitability with potential impacts on biodiversity and ecosystem function.

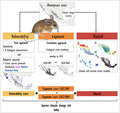

## 1 INTRODUCTION

Anthropogenic climate change (CC) is a major threat to biodiversity (Habibullah et al., 2022; Nunez et al., 2019), and several studies have proposed different approaches for identifying species at risk (Foden et al., 2009; Foden & Young, 2016; Jones & Cheung, 2018; Pacifici et al., 2018; Young et al., 2011). The impact of CC on species has been mainly evaluated using a trait‐based, correlative, or mechanistic approach (Pacifici et al., 2018). A trait‐based approach includes species biological characteristics to identify sensitivity (Sandin et al., 2014) (see below). The correlative approach has included projecting changes in climatic suitability of species potential distributions based mostly on ecological niche modeling (Aubin et al., 2016; Peterson et al., 2011). A mechanistic approach is a process‐based model projecting species' responses to changing environmental conditions by incorporating biological processes, thresholds, and interactions (Keith et al., 2014; Ureta et al., 2012, 2018). These mechanistic models use information on the species physiology and demography to provide detailed information of the processes that increase risks under CC scenarios (Chown et al., 2010; Ureta et al., 2012).

An alternative is to use a combination of these methodological approaches (Albouy et al., 2020; Foden et al., 2009; Leclerc et al., 2020; Zhang et al., 2019). Previous studies have used this combined approach through vulnerability indexes based on the old conceptual framework proposed by the Intergovernmental Panel on Climate Change (IPCC), in which species vulnerability is defined as its exposure, sensitivity, and adaptive capacity (Solomon et al., 2007). However, the latest conceptual framework of the IPCC (2014, 2020, 2021b, 2022) defines species risk as a function of vulnerability (sensitivity and adaptive capacity), exposure, and hazard. Sensitivity is defined by the degree of direct or indirect impact of CC on a species (or another system), and adaptive capacity is defined as the ability of a species (or another system) to cope with CC (IPCC, 2014, 2022). Exposure is defined as the presence of species (or other systems) in areas that could be detrimental due to CC (IPCC, 2014, 2022). Hazard is defined as an occurrence or a potential tendency of a physical event to occur that could cause harm to species (or any system). Thus, there is a need for a uniform conceptual framework to allow comparisons of studies that evaluate the impacts of CC on biodiversity and ecosystems. To our knowledge, there is only one study aimed to evaluate risk in species‐rich areas due to CC applying the latest IPCC conceptual framework (Pacifici et al., 2018); however, that study did not identify species at higher risk.

In this study, we used the latest conceptual framework of the IPCC to project future risk for species, taxonomic, and functional groups of terrestrial mammals occurring in Mexico. The taxonomic diversity was evaluated at the order level of these species. The functional diversity included species grouped with similar ecological functions, such as food habits (trophic level), movement and dispersal characteristics (locomotion), and biological characteristics (body size), without considering evolutionary lineages (Duckworth et al., 2000). If species in a taxonomic order or species in functional groups are at high risk, we should expect associated conservation threats on biodiversity, phylogenetic diversity, ecosystem functioning, and provision of environmental services (Barnes et al., 2017; Memmott et al., 2007). We also incorporated current and future land‐use/cover change (LUCC) scenarios (Mendoza‐Ponce et al., 2018) (see below) into the exposure component to integrate both major factors (CC and LUCC) of biodiversity loss and ecosystem degradation into our analyses (Barnes et al., 2017; Buizer et al., 2014; Linero et al., 2020).

We included the terrestrial mammals given that it is a well‐studied group playing fundamental ecological roles (Lacher et al., 2019) and because Mexico is exceptionally diverse in this group, hosting 463 species (12% of total worldwide), of which 30% of species are endemic (CONABIO, 2020). Furthermore, the taxonomic status (order of mammals) of most species has been thoroughly studied (Ramírez‐Bautista et al., 2020; Sánchez‐Cordero et al., 2014), and species assignment to functional groups of terrestrial mammals has been previously proposed (Arita & Rodríguez, 2004; Arnold, 1983; Eisenberg & Redford, 1989; Gómez‐Ortiz & Moreno, 2017; González‐Salazar et al., 2014; González‐Suárez et al., 2013; Lacher et al., 2019; Medellín, 1993; Nowak & Walker, 1999; Robinson & Redford, 1986; Violle et al., 2007). We used functional groups to describe their role in ecosystems (Arnold, 1983; Lacher et al., 2019; Violle et al., 2007). We considered the following functional groups: trophic group, body size, and locomotion group. For instance, a trophic group is a functional trait associated with food resources, population dynamics, pollination, seed dispersal, and trophic plasticity, among other characteristics (Lacher et al., 2019). Body size is a functional trait associated with a demand for trophic resources, energy expenditure, and energy flow between trophic levels (Lacher et al., 2019), and it is highly correlated with mammal life‐history traits (Stearns, 1983). Locomotion traits are associated with spatial resource use and habitat adaptations for foraging and refuge (Gómez‐Ortiz & Moreno, 2017). The advantage of using these functional traits is that they are complementary and non‐redundant, and these categories have been previously used in studies on the functional diversity of mammals (Munguía et al., 2016) and other terrestrial vertebrates (Gómez‐Ortiz & Moreno, 2017) in Mexico.

Our aims were to (1) build a species risk index (as a function of vulnerability, exposure, and hazard) to project risks for species, taxonomic, and functional group diversities of terrestrial mammals under two contrasting general circulation models (GCMs) and future CC and LUCC projections and (2) identify cross‐time shifts in climatic suitability projections of species‐rich areas in potential changes in species composition and turnover.

## 2 MATERIALS AND METHODS

### 2.1 Trait‐based approach

**FIGURE 1:**
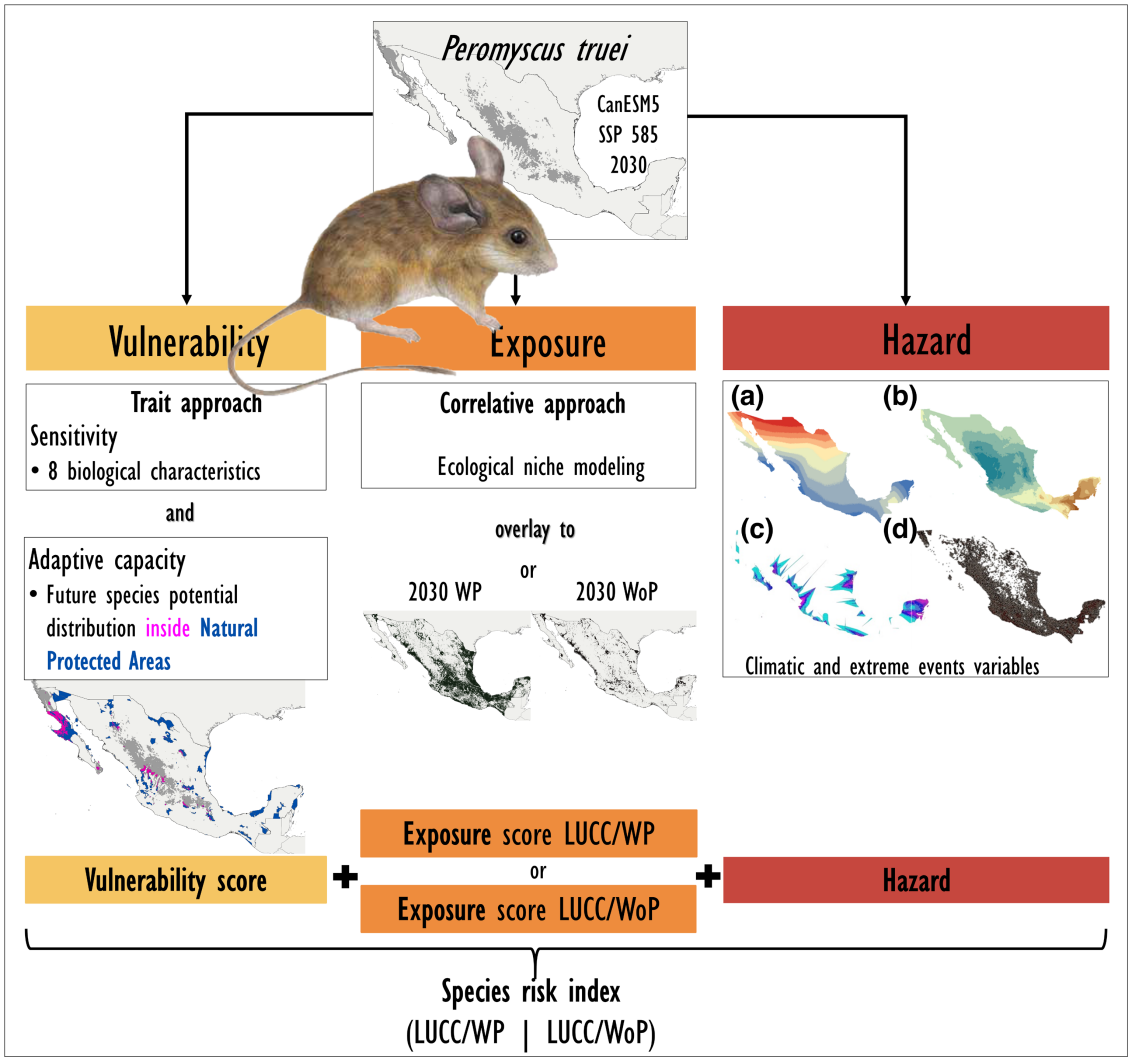
Flowchart depicting an example with the pinyon mouse, *Peromyscus truei*, to illustrate the methodological approach. Top: Species potential distribution in Mexico. Middle: Sequence of analyses to building a projected species risk index, where risk is a function of vulnerability (sensitivity and adaptive capacity), exposure, and hazard (IPCC, 2014). First, we show a trait‐based approach for estimating the sensitivity (including eight species biological characteristics) and adaptive capacity (overlap of future species potential distribution with protected areas) of the species vulnerability score. Then, we used a correlative approach using ecological niche models to project future species potential distribution for 2030, under the CanESM5 and SSP5‐RCP 8.5 climate change (CC) scenario for estimating the species exposure score. The exposure score land‐use and cover change/with precautionary principle (LUCC/WP) assumes that agricultural areas are unsuitable habitats, and the exposure score LUCC/without precautionary principle (WoP) assumes that agricultural areas are suitable habitats for this species, under projected future LUCC scenarios. The hazard species risk score was estimated by overlapping extreme events on species future potential distribution, as annual temperature change between the evaluated and present‐day scenarios (a), precipitation change between the evaluated and present‐day scenarios (b), historic observation of hurricanes intensity (c), and historic observation of fires (d). Bottom: The vulnerability, exposure (LUCC/WP and LUCC/WoP), and hazard risk scores were added to estimate the species risk index LUCC/WP and species risk index LUCC/WoP. The same sequence of analyses was produced for two GCM (CanESM5 and BCC‐CSM2.MR), three cross‐time periods (2030, 2050, and 2070), and two scenarios (SSP2‐RCP4.5 and SSP5‐RCP8.5) for all terrestrial species included in this study. See “Section 2” for details. GSM, general circulation model.

We assessed sensitivity using a trait‐based approach (Sandin et al., 2014) with the following biological characteristics: number of ecoregions in which a species occurred as a proxy of ecological plasticity, species conservation status according to categories assigned by the IUCN Red List (IUCN, 2021), population status (increasing, stable, or decreasing), endemism, feeding habits (specialist or generalist), dependency to responding to environmental cues, dispersion ability, and a restricted distribution on islands. Most of the biological information was obtained from scientific literature and experts' opinions (Appendices S1 and S2). Species' biological characteristics were then scored as positive, negative, or neutral (see Appendices S1 and S2 for details). For each variable, a species ranking was assigned (ranging from 1 to 450; e.g., in case of no species repeating values). A high species risk corresponded to a high rank value. We added the ranking values from each biological characteristic and a final ranking was assigned (Figure 1).

### 2.2 Correlative approach

We used ecological niche modeling to assess species exposure and adaptive capacity to CC by projecting species potential distribution using unique species records and 19 bioclimatic variables (~10 km^2^) from the WorldClim database (Fick & Hijmans, 2017). From the 19 bioclimatic variables, we selected for each species those that presented less collinearity (see S‐codes). We also used all 19 bioclimatic variables to search possible extrapolations with the ExDet tool. For each species, we used the “corrSelect” function of the fuzzySim package (Barbosa, 2015) to conduct a Pearson correlation; pre‐selected variables with thresholds >0.8 were included in the model. Only variables that were not strongly correlated (threshold <0.8) were included in the model. Of a total of 463 terrestrial mammals occurring in Mexico (CONABIO, 2020; SEMARNAT, 2010), we evaluated 450 species holding 25 or more unique records. We only included species with a minimum of 25 unique records for our modeling approach, given that it has been demonstrated that this threshold is useful to generate robust distribution models (Pearson et al., 2007). The occurrence records for 330 species were obtained from the National System of Biodiversity Information (SNIB, 2020). For the remaining 120 species holding <25 unique records, we obtained point occurrences based on the IUCN polygons (IUCN, 2021; Alhajeri & Fourcade, 2019). To avoid spatial bias and correlation between point occurrences for species modeled, we set 10 km as the minimum distance between them (Pearson et al., 2007). For endemic species with restricted distributions even in IUCN polygons, we reduced the minimum distance to 5 km between point occurrences to reach a minimum of 25 unique records. In this case, we assumed that spatial independence between occurrences could be obtained with a reduced minimum distance. For species holding >300 unique records from the SNIB database, we reduced our sample by estimating the average distance between the two nearest records with the remaining point occurrences. Those records with the shortest distance were discarded until reducing our number to a maximum of 300. A high number of records can generate problems associated with spatial bias in the modeling (Aiello‐Lammens et al., 2015). For species holding >300 unique records, the number of records between species differed by several orders of magnitude, for example, it is not equivalent to randomly choosing 300 records from a 10,000 sample than from a 1000 sample. In this case, the number of point occurrences were randomly taken for each species using an exponential equation (Appendix S1—codes) of which parameters depended on the maximum (300) and minimum (25) number of unique records. The number of point occurrences reached 300 as the number of records in the sample increased.

We used the BIOMOD platform that facilitates the ensemble of several algorithms (Thuiller et al., 2009). The calibration and transference areas were determined by the intersection between the ecoregions, including at least one‐point occurrence of a species, and a 3° buffer surrounding the localities. Then, a 2° buffer was created around the intersecting area. We considered a limited full‐dispersal assumption (Peterson et al., 2001), where species show full‐dispersal ability restricted to the corresponding calibration and transference areas. Thus, we included the species dispersal abilities in the modeling exercise. Given that some species might not be able to disperse and establish in new climatically suitable areas, we also calculated the area loss projected under a non‐dispersal assumption as a reference for comparisons (Peterson et al., 2001). The final species risk index only considered a limited full‐dispersal assumption (Appendices S2 and S7).

We used different algorithms that maximized their performance with 10 replicates and 1000 pseudo absences (randomly selected): GLM, GAM, CTA, and RF (Barbet‐Massin et al., 2012). We incorporated GBM and Maxent because both algorithms proved robust predictive performance in models of 20 randomly selected species. We used 70% and 30% of records for model calibration and validation, respectively, and evaluated the models with Kappa, TSS, and ROC. We made each individual algorithm map binary by maximizing the TSS value. Then, we developed a weighted ensemble map (considering the AUC value) for each species at each evaluated scenario and time for all replicates of all algorithms (Figure 1; Appendix S3).

To project the impact of CC on species potential distributions, we used two contrasting GCM: BCC‐CSM2.MR (BCC) and CanESM5 (CAN), with two shared socioeconomic pathways (SSP) that incorporate demographic trends, social, technological, economic patterns, and developments (Riahi et al., 2017): SSP2‐RCP 4.5 (245) and SSP5‐RCP 8.5 (585). GCMs are models representing physical processes in the atmosphere, ocean, cryosphere, and land surface, and they are considered the most robust approximation to simulate anthropogenic CC (IPCC, 2021a). The CanESM5 circulation model has shown high performance at representing the current climate in Mexico (Altamirano del Carmen et al., 2021). These two GCMs project different future climates for Mexico; the CAN projects warmer conditions and higher precipitation (1.943–5.204°C and −77.29–16.289 mm), and the BCC.MR model projects cooler and drier conditions (1.199–3.37°C and −86.826 to −8.463 mm) (Appendix S2). Each GCM has two possible socioeconomic pathways. The first model 245 represents a “middle of the road” narrative, where socioeconomics trends do not shift markedly from historical patterns, and CO_2_ decreases in the mid‐century although it does not reach net zero emission by 2100. This model globally projects temperature rises of 3°C by the end of the century. The 585 is a “fossil‐fueled development” narrative, where socioeconomic growth is dependent on abundant fossil fuel resources, CO_2_ emissions would double in 2050, and average global temperature will increase over 4.5°C (IPCC, 2021a; Riahi et al., 2017).

**FIGURE 2:**
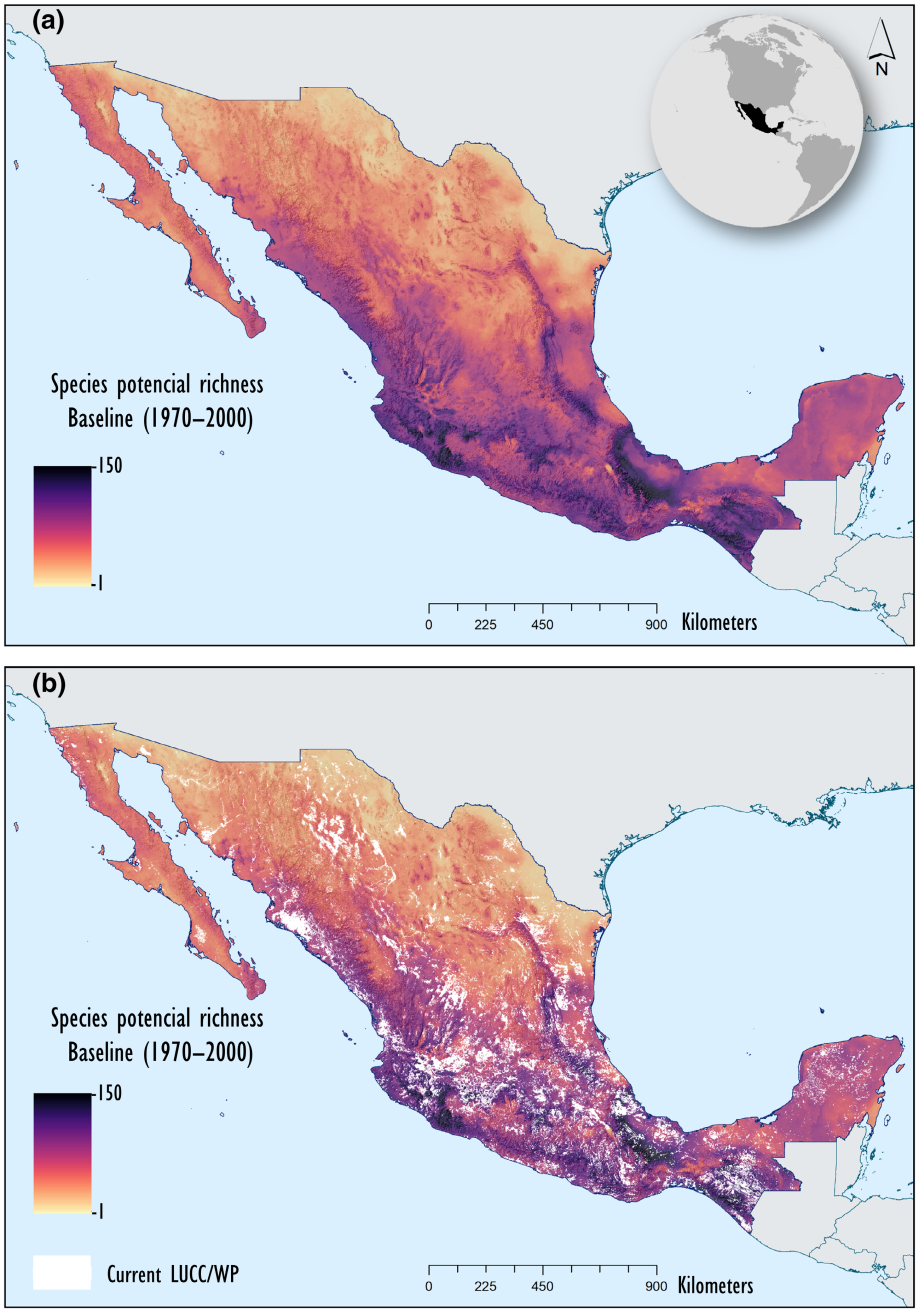
Projected species‐rich areas under scenario 1970–2000. Cumulative binary maps of all evaluated species of terrestrial mammals were projected to identify geographic areas with climatic suitability holding high species richness. (a) Maps projecting climatic suitability for species‐rich areas excluding future land use and cover changes/with precautionary principle (LUCC/WP; areas in dark colors) and (b) including LUCCs (LUCC/without precautionary principle [WoP]; areas in white). Scale in colored bar indicates number of species.

**FIGURE 3:**
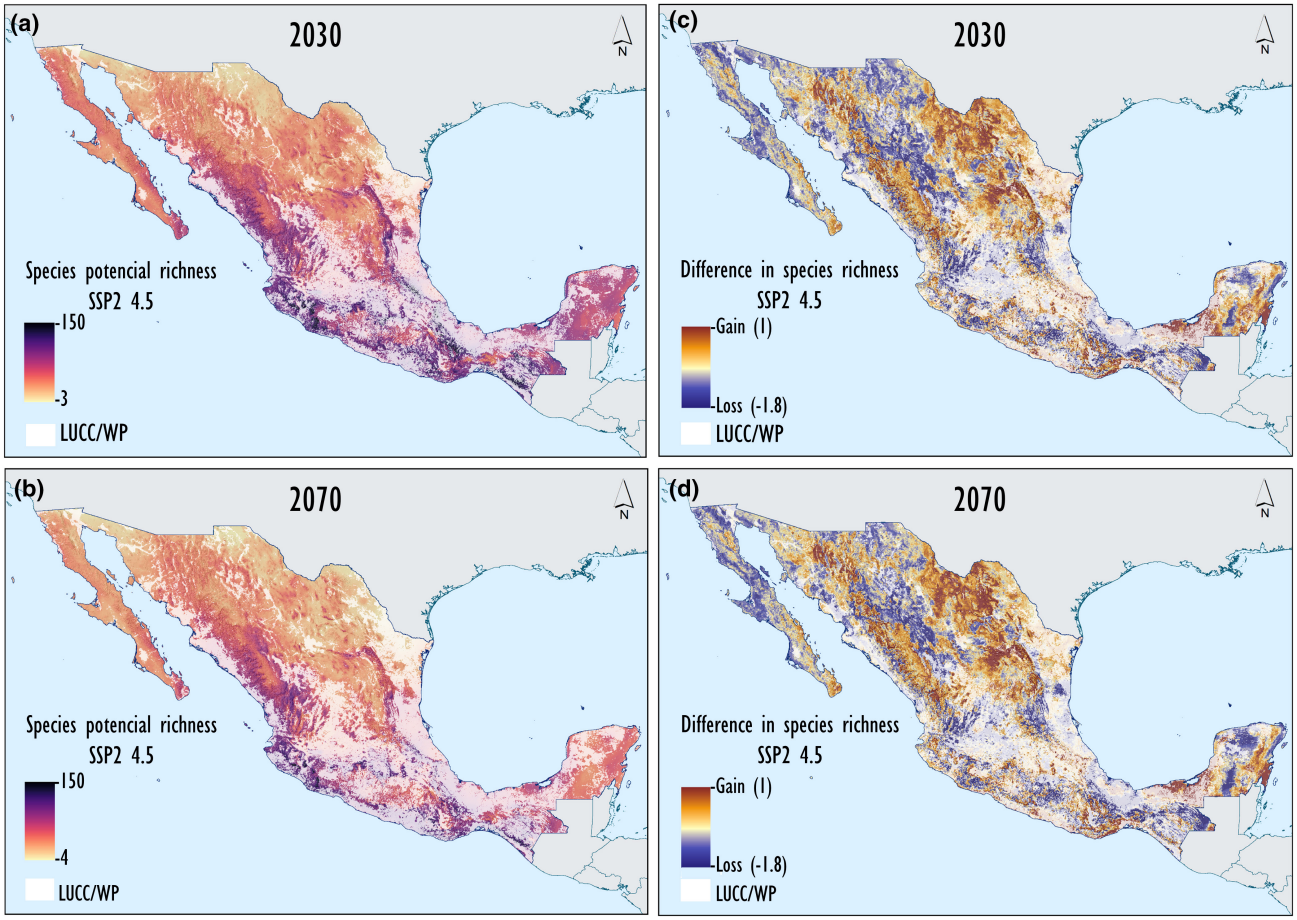
Maps depicting species‐rich areas and changes in species‐rich areas (gain and loss) projected for 2030 (a, b), and 2070 (c, d), respectively. Suitable areas under climatic and land‐use and cover change (LUCC) scenarios, assuming rainfed agricultural areas as unsuitable habitats for species (LUCC/with precautionary principle [WP]). Geographic areas with higher changes in climatic suitability (c, d), including the GCM CanESM5 under the climate change (CC) scenario SSP5‐RCP 8.5 (worst case scenario). See “Section 2” for details and MS 1 for GCM BCC‐CSM2.MR.

We conducted our cross‐time CC projections for 2030 (2021–2040), 2050 (2041–2060), and 2070 (2081–2100) using a climate raster at 2.5 min resolution (Fick & Hijmans, 2017). Because we examined species responses under two GCMs with their corresponding SSPs (representing a pessimistic and a mid‐optimistic scenario) under 3‐year projections (2030, 2050, and 2070) (Appendix S2), a total of 12 combinations of CC scenarios were evaluated. We also identified projections of species‐rich areas under these combinations of CC scenarios, based on the cumulative projections of species potential distributions under current and CC scenarios (2030, 2050, and 2070) and quantified species‐rich area changes by subtracting current from potential CC species‐rich areas. Species‐rich areas identify species “hotspots” of biodiversity as potential future prioritization conservation areas. Furthermore, differences in species‐rich areas showing areas with an increase (gain) or decrease (loss) in suitability for species were used as projections to identify geographic regions where higher species turnover can be expected (Figures 2 and 3).

We overlaid species‐rich areas and areas with higher species‐rich differences with future LUCC scenarios. The LUCC scenarios were developed in Dinamica EGO (version 3.0.17.0) and obtained from Mendoza‐Ponce et al. (2018). Their model includes the following: (1) the definition of the land‐use and cover categories and the calculation of transition matrices, (2) the categorization of continuous variables, (3) estimations of the statistical weights of the explanatory variables, (4) analyses of the correlation between variables, and (5) a short‐term simulation to validate the model and long‐term projections under different trajectories into which the socioeconomic and the CC scenario were incorporated (see Appendix S2 for detail in their methods). The LUCC scenarios were independently validated by comparisons with observed maps from 2011 and 2015. Long‐term projections and scenarios were produced by combining socioeconomic and climatic variables and land‐use and cover transition rates (see Mendoza‐Ponce et al., 2018 and Appendix S1 for details). The period selected for model calibration was 1993–2011. LUCC scenarios used in our study were developed before CMIP6 was released (e.g., with GCMs: CNRMC M5, GFDL CM3, MPI‐ESMLR, and HADGEM2). In our species niche modeling, we used CAN as the GCMs given its robust performance to simulate the climatic system of the Northern Hemisphere. Thus, we used the LUCC scenario built with HADGEM2 (HAD) because it has been evaluated to be similar in numerical approximations to CAN (Knutti et al., 2013).

The LUCC scenario layers from Mendoza‐Ponce et al. (2018) used in our study represented population medium fertility, mortality, and migration trends (O'Neill et al., 2017), and economic moderate development (although there are significant heterogeneities nationwide), and trends in land‐use and cover changes fell into historic average projections. To incorporate these trends, Mendoza‐Ponce et al. (2018) calculated all rates of historical land‐use and cover maps by combining the available national maps and using the Food and Agriculture Organization (1995) equation to estimate deforestation. An average‐tendency scenario was plausible to combine with optimistic and pessimistic scenarios as they do not involve additional assumptions; rather, they are based on historical trends.

### 2.3 Species risk index

Our proposal defined a species risk index based on the latest IPCC framework (IPCC, 2021b; Figure 1). We obtained a ranking value for each variable of the risk index from the vulnerability, exposure, and hazard components (Table 1). In case several species had the same ranking for a specific variable, we assigned an average ranking value. Once rankings were assigned to all variables, they were first added in each component and then added from the three components (vulnerability, exposure, and hazard) into a final risk value. The final risk value was standardized (each value divided by the maximum value of the variable) to obtain risk values between 0 and 1 for facilitating interpretation (Figures 1, 2, 3; Table 1).

**TABLE 1:** List of variables selected for calculating the species risk index

Variables	Variables description
Sensitivity Score	Ranking obtained from the biological characteristics of the species
NPA	Percentual change of pixels inside a PA between present‐day scenario and the future scenario evaluated
NPA Score	NPA ranking
Vulnerability Score	Sum of the biological sensitivity and the NPA Score
Current PDA vs. Projected PDA	Percentual PDA change between present‐day scenario and the CC scenario evaluated
PDA Score	PDA ranking
Relative LUCC/WP	Percentage of PDA area inside a not viable area given projected LUCC excluding rainfed agriculture and cattle grassland
LUCC/WP Score	LUCC/WP ranking
Relative LUCC/WoP	Percentage of PDA area inside a not viable area given projected LUCC including rainfed agriculture and cattle grassland
LUCC/WoP Score	LUCC/WoP ranking
Exposition WP Score	Sum of PDA weight and LUCC/WP Score
Exposition WoP Score	Sum of PDA weight and LUCC/WoP Score
T°Change	Mean annual temperature absolute change between present‐day and CC scenarios
T°Change Score	T°Change ranking
P Change	Annual precipitation absolute change between present‐day and CC scenarios
P Change Score	P Change ranking
Fires	Number of fires relative to the PDA in the scenario evaluated
Fires Score	Fires ranking
Hurricanes	Hurricane intensity relative to the PDA in the scenario evaluated^a^ ^,^ ^b^
Hurricanes Score	Hurricanes intensity ranking
Hazard Score	Sum of T°Change Score, Fires Score, and Hurricanes Score
Species risk score WP	The sum of vulnerability, exposition, and hazards scores with LC/WP Score
Risk normalization	The normalization by the maximum number of Risk Score WP to obtain values between 0 and 1
Species risk score WoP	The sum of vulnerability, exposition, and hazards scores with LC/WoP Score
Risk normalization	The normalization by the maximum number of Risk Score WoP to obtain values between 0 and 1

*Note*: Higher risk values are expected for species with higher risk.

Abbreviations: CC, climate change; LUCC, land‐use and cover change; PA, protected area; WoP, without precautionary principle; WP, with precautionary principle.

^a^ Average of the category and frequency of hurricanes in a 10 km^2^ cell.

^b^ The weighted mean was given by the percentage of potential distribution area (PDA) overlaying specific hurricane intensity. See Appendix S3 for further details.

The first ranking was obtained from sensitivity (trait‐based approach; see above), followed by the species adaptive capacity, estimated by comparing the percentage of pixels of climatic suitability inside protected areas under a present‐day scenario (CONANP, 2021), and evaluated under the 12 combinations of CC scenarios (Appendices [Link], [Link], [Link]). We assumed that protected areas with minimum LUCC provide adequate environmental conditions for species to best cope with CC (Peach et al., 2019). We added the sensitivity ranking with the adaptive capacity ranking to obtain the vulnerability score; a higher vulnerability corresponded with higher values (Appendices S1 and S4).

We evaluated the exposure component using the correlative approach (see above). The exposure component included two main variables (Tables 2 and 3). The first variable was obtained by quantifying changes in climatic suitability between current and the 12 combinations of CC scenarios. We calculated a corresponding ranking by assigning higher ranking values to higher reductions in climatic suitability. Then, we calculated the number of pixels of suitable climate conditions in each CC combination inside a non‐suitable habitat of LUCC (Mendoza‐Ponce et al., 2018), relative to the total number of pixels of suitable climate conditions. We assumed two potential species responses to future LUCC scenarios: (1) excluding rainfed agriculture areas and grassland for cattle as species suitable habitats*—*“with precautionary principle” (LUCC/WP)*—*and (2) including rainfed agriculture areas and grassland for cattle as species suitable habitats—“without precautionary principle” (LUCC/WoP)—(Linero et al., 2020; Mayani‐Parás et al., 2021) (Table 3). Some studies have observed that small mammals can show resident populations in rainfed agricultural areas and cattle grasslands (Pekin & Pijanowski, 2012; Riojas‐López et al., 2018; Schieltz & Rubenstein, 2016). However, large‐to‐medium‐sized terrestrial mammals face challenging conservation conditions to maintain resident and reproductive populations in rainfed agricultural areas and cattle grasslands (Hidalgo‐Mihart et al., 2019; Ren et al., 2021; Schieltz & Rubenstein, 2016). We argue that contrasting both options (LUCC/WP and LUCC/WoP) in projections of species potential distributions provides a better understanding of how future scenarios of habitat loss will impact species of terrestrial mammals (Linero et al., 2020). For each variable, a ranking value was assigned where a higher number of pixels inside a non‐suitable habitat of LUCC relative to the total number of pixels in the corresponding combination of CC evaluated had higher ranking values. We obtained two final exposure risk scores: exposure risk score LUCC/WP and exposure risk score LUCC/WoP (Figure 1; Table 1).

**TABLE 2:** Species potential distribution (mean, median, and range)

SPD limited full dispersal						
		245 BCC	245 CAN	585 BCC	585 CAN	Range
2030	Mean	0.072	0.064	0.074	0.066	0.064–0.074
	Median	0.060	0.082	0.053	0.077	0.053–0.082
2050	Mean	0.131	0.088	0.168	0.114	0.088–0.168
	Median	0.138	0.130	0.204	0.199	0.130–0.204
2070	Mean	0.124	0.105	0.166	0.167	0.105–0.167
	Median	0.138	0.177	0.272	0.375	0.138–0.375

SPD non‐dispersal						
2030	Mean	0.227	0.259	0.251	0.271	0.227–0.271
	Median	0.172	0.206	0.188	0.218	0.172–0.218
2050	Mean	0.312	0.327	0.380	0.385	0.312–0.385
	Median	0.268	0.278	0.339	0.345	0.268–0.345
2070	Mean	0.353	0.370	0.456	0.498	0.353–0.498
	Median	0.294	0.330	0.432	0.502	0.294–0.502

*Note*: Species potential distributions were estimated for all GCM and climate change (CC) scenarios for 2030, 2050 and 2070 for the terrestrial vertebrates occurring in Mexico. SPD = species potential distributions; 245 BCC, 245 CAN, 585 BCC, and 585 CAN = CC models included in the analysis. SPD were estimated under a limited full‐dispersal assumption, and a non‐dispersal assumption. See “Section 2” for details.

Abbreviation: GCM, general circulation model.

**TABLE 3:** Species potential distribution inside suitable habitats of land‐use/cover change (LUCC) scenarios (mean, median, and range)

Limited full dispersal						
LUCC/WP						
		245 BCC	245 CAN	585 BCC	585 CAN	Range
2030	Mean	0.394	0.395	0.391	0.392	0.391–0.395
	Median	0.436	0.431	0.432	0.431	0.431–0.436
2050	Mean	0.430	0.424	0.424	0.424	0.424–0.43
	Median	0.479	0.472	0.479	0.473	0.472–0.479
2070	Mean	0.454	0.451	0.447	0.451	0.447–0.454
	Median	0.510	0.510	0.503	0.500	0.500–0.510

LUCC/WoP						
2030	Mean	0.081	0.080	0.079	0.080	0.079–0.081
	Median	0.079	0.080	0.078	0.078	0.078–0.08
2050	Mean	0.090	0.090	0.087	0.087	0.087–0.09
	Median	0.088	0.088	0.083	0.085	0.083–0.088
2070	Mean	0.099	0.098	0.093	0.088	0.088–0.099
	Median	0.095	0.094	0.089	0.085	0.085–0.095

Non‐dispersal						
LUCC/WP						
2030	Mean	0.398	0.401	0.398	0.401	0.398–0.401
	Median	0.438	0.436	0.439	0.438	0.436–0.439
2050	Mean	0.434	0.438	0.431	0.439	0.431–0.439
	Median	0.480	0.488	0.477	0.486	0.477–0.488
2070	Mean	0.462	0.468	0.460	0.471	0.460–0.471
	Median	0.516	0.524	0.511	0.515	0.511–0.524

LUCC/WoP						
2030	Mean	0.084	0.085	0.083	0.084	0.083–0.085
	Median	0.079	0.080	0.078	0.079	0.078–0.080
2050	Mean	0.096	0.098	0.095	0.097	0.095–0.098
	Median	0.087	0.089	0.084	0.088	0.084–0.089
2070	Mean	0.109	0.109	0.105	0.106	0.105–0.109
	Median	0.095	0.098	0.096	0.094	0.094–0.098

*Note*: Calculations were estimated for all GCM and climate change (CC) scenarios for 2030, 2050, and 2070 for the terrestrial vertebrates occurring in Mexico. LUCC/WP = species potential distribution inside unsuitable habitats for species with precautionary principle (rainfed agricultural areas and cattle grassland are not suitable habitats for species). LUCC/WoP = species potential distribution inside unsuitable habitats for species without precautionary principle (rainfed agricultural areas and cattle grassland are suitable for the species). 245 BCC, 245 CAN, 585 BCC, and 585 CAN = CC models included in the analysis. Limited full dispersal assumed that species showed full‐dispersal ability restricted to the corresponding calibration and transference areas and non‐dispersal assumed that species were unable to disperse to new areas showing suitable climatic conditions. See “Section 2” for details.

Abbreviation: GCM, general circulation model.

We evaluated the hazard component using the projected changes in the mean annual temperature and annual precipitation compared to current conditions and the observed tendency in the number of fires and hurricanes relative to the total suitable climatic area in the corresponding combination of the evaluated CC scenarios. Regarding the temperature and precipitation, species found suitable climatic conditions in the combinations of evaluated CC scenarios (inside their climatic range). However, areas showing strong changes in climate affect environmental conditions resulting in unknown ecological consequences (Prieto‐Torres et al., 2021). We also evaluated the number of fires and hurricane intensity occurring in the projections of species potential distributions (Figure 1). Hurricanes and fires were obtained from observational data (1970–2015 and 2001–2019, respectively) (GRDP, 2021; NASA, 2021). Hurricane intensity was calculated by the average category and frequency of hurricanes present in a 10 km^2^ cell. We obtained a ranking value for these four variables (high mean annual temperature changes, high mean annual precipitation changes, fires, and hurricanes) (Table 1) and added these ranking values into a final hazard score for each species.

The species risk index was estimated by adding its vulnerability, exposure, and hazard components final scores. Higher ranking values indicated higher species risk. All variables were multiplied by −1 when showing a negative relationship with risk (LUCC, mean annual temperature change, precipitation, number of fires, and hurricane intensity) (Figure 1; Appendices S2 and S4). Given that we obtained two different exposure scores (LUCC/WP and LUCC/WoP), we also estimated two species risk indexes (species risk index LUCC/WP, and species risk index LUCC/WoP) for each species (Figure 1; Table 4). Lastly, we statistically compared the central tendency between groups of terrestrial mammals using a Wilcoxon test (Appendices S5 and S6). We ranked the species by their risk score and obtained the top decile of highest species risk under all combinations of CC scenarios (Appendices S2 and S4) to account for the variability within groups, and allowing comparison between all evaluated groups. Thus, we were able to detect the taxonomic orders and functional groups that frequently occurred in the top decile of highest species risk.

**TABLE 4:** Number of species with highest risk scores (ranges) by functional and taxonomic groups of terrestrial mammals

	*N*	2030		2050		2070	
		Species risk score LC/WP	Species risk score LC/WoP	Species risk score LC/WP	Species risk score LC/WoP	Species risk score LC/WP	Species risk score LC/WoP
Trophic group							
Frugivore–granivore	26	11.54–23.08	15.38–23.08	15.38–19.23	15.38–19.23	11.54–19.23	11.54–19.23
Herbivore–browser	9	11.11–22.22	11.11–22.22	11.11–11.11	11.11–11.11	0.00–11.11	0.00–11.11
Insectivore under canopy	56	16.36–23.64	16.36–23.64	18.18–23.64	18.18–23.64	20.00–29.09	20.00–29.09
Myrmecophage	3	0.00	0.00	0.00–33.33	0.00–33.33	0.00–33.33	0.00–33.33
Piscivore	7	14.29–28.57	14.29–28.57	0.00–28.57	0.00–28.57	0.00–28.57	0.00–28.57
Body mass							
Large	17	11.76–11.76	11.76–11.76	5.88–17.65	5.88–17.65	5.88–17.65	5.88–17.65
Medium–large	34	8.82–14.71	8.82–14.71	8.82–11.76	8.82–11.76	5.88–14.71	5.88–14.71
Locomotion							
Arboreal	16	37.50–50.00	37.50–0.00	37.50–43.75	37.50–43.75	25.00–31.25	25.00–31.25
Semi‐aquatic	13	15.38–7.69	15.38–23.08	7.69–23.08	7.69–23.08	7.69–23.08	7.69–23.08
Order							
Artiodactyla	9	11.11–22.22	22.22	11.11–22.22	11.11–22.22	11.11–22.22	11.11–22.22
Cingulata	2	0.00–50.00	0.00	0.00–50.00	0.00–50.00	0.00–50.00	0.00–50.00
Didelphimorphia	8	25.00–37.50	25.00–50.00	25.00–37.50	25.00–37.50	0.00–25.00	0.00–25.00
Eulipotyphla	34	11.76–29.41	20.59–23.53	11.76–29.41	11.76–29.41	14.71–38.24	14.71–38.24
Perissodactyla	1	0.00–100.00	0.00–0.00	0.00–100.00	0.00–100.00	0.00–100.00	0.00–100.00
Lagomorpha	11	18.18–27.27	18.18–27.27	9.09–18.18	9.09–18.18	9.09–18.18	9.09–18.18
Primates	3	33.33	33.33	33.33	33.33	33.33	33.33

*Note*: We included all possibilities from all general circulation models (GCMs) and scenarios projected for 2030, 2050, and 2070. Groups with the highest % of their species in top decile of any time (land‐use and cover change/with precautionary principle LUCC/WP or LUCC/without precautionary principle [WoP]) are shown in the table. *N* = number of species corresponding to each trophic and taxonomic group. Species risk score LUCC/WP assumed rainfed agriculture areas as unsuitable habitats for species. Species risk score LUCC/WoP assumed rainfed agriculture areas as suitable habitat for species. See “Section 2” for details.

## 3 RESULTS

A total of 15 species lost their entire area of suitable climatic conditions at least under one of the combinations of CC scenarios. Even though these species were the most exposed according to our proposed risk index, we were not able to calculate their final risk score as the loss of the suitable conditions equaled 100%. Species that have been rarely recorded in Mexico but did not show suitable climatic conditions under the historical conditions (1970–2000) were *Eumops hansae* and *Microtus pennsylvanicus*. Species that lost their entire climatic suitability under any future CC scenarios were *Centronycteris centralis*, *Cratogeomys fulvescens*, *Cryptotis goodwini*, *Dipodomys deserti*, *Geomys tropicalis*, *M. umbrosus*, *Peromyscus guardia*, *P. hooperi*, *P. nasutus*, *P. polius*, *Reithrodontomys spectabilis*, *Sorex ornatus*, and *Tamiasciurus mearnsi* (Appendix S3). Most of these species were strongly sensitive to CC (Appendix S2), showing an average sensitivity value of 350 (range, 216–450) (Appendix S1). These species were included in the projected species‐rich areas under current and all CC combinations (Appendix S2). Species that were frequently present in the top decile of greatest risk were the primates *A. palliata* and *A. pigra*, the bat *Balantiopteryx io* and the marsupials *Chironectes minimus* and *Caluromys derbianus*.

Species potential distributions lost from 20% of projected suitable climatic conditions in 2030 (2021–2040) to almost 40% in 2070 (2081–2100) under the SSP 585 CanESM5 scenario, assuming limited full dispersal. Species lost an additional 39.1%–51% of projected suitable habitat due to LUCC/WP under both GCMs. Under a non‐dispersal assumption of species to reach areas holding new suitable climatic conditions is projected to be at a loss of 50.2% of their projected climatically suitable conditions by 2070 (2081–2100) (Tables 2 and 3). Furthermore, projected species‐rich areas holding >75 species occurred in the mountain ranges of western Mexico. Projected differences in species‐rich areas were maintained in the Sierras regions in western Mexico but showed a reduction in the Transvolcanic Belt in central Mexico due to continued LUCC (Figures 2 and 3). Under a species limited full‐dispersal assumption, potential species‐rich areas showed a maximal reduction of 36.4% due to projected unsuitable climatic conditions, and 57.1% of maximal reduction by 2070, when adding projected unsuitable LUCC/WP and LUCC/WoP, respectively (Table 4; Appendix S2). Under a species non‐dispersal assumption, potential species‐rich areas showed a maximal reduction of 50.2% due to projected unsuitable climatic conditions and of 52.4% when adding projected unsuitable LUCC/WP and LUCC/WoP, respectively. Cross‐time shifts in species‐rich areas increased in number of species (gain) in projected climatic suitability in northeast and southeast and decreased in number of species (loss) in northwest and southern Mexico (Figure 3).

Most differences between groups were not significant (Appendix S5). Thus, we showed the results of the taxonomic and functional groups that were more frequent in the top decile of highest risk as ranges to include all combinations of GCMs and SSPs (Table 4). Of the 11 taxonomic orders occurring in Mexico that included eight or more species, Eulipotyphla had highest risk scores followed by Didelphimorphia, Artiodactyla, and Lagomorpha (Table 3). Of the 19 trophic groups that included three or more species, piscivores had highest risk scores followed by insectivores under canopy, frugivores–granivores, browsing herbivores, and myrmecophagous. Of the five body‐sized functional groups, large‐ and large‐to‐medium‐sized species had higher risk scores than small‐sized species. Of the seven locomotion groups, arboreal and semi‐aquatics had highest risk scores (Table 4; Appendix S7). The American beaver, *Castor canadensis*, ranked highest by belonging to more functional groups at risk, including herbivore–browser, semi‐aquatic, and large‐to‐medium‐sized body (Appendices S2 and S4).

## 4 DISCUSSION

We used a novel protocol to assign projected species risk scores for the terrestrial mammals occurring in Mexico under future CC and LUCC. Our study incorporated potential impacts at species, taxonomic, and functional group diversities using a combined trait‐based (species biological characteristics) and correlative (ecological niche modeling projected as species potential distributions) approach to quantify the vulnerability, exposure, and hazard components (species risk index) proposed by IPCC (2021b) (Figure 1).

### 4.1 Risk in species diversity

We assigned all species a risk score, except for the 15 species projected to lose their entire suitable climatic conditions in Mexico under all combinations of evaluated CC scenarios. Moreover, species showing highest risk scores due to drastic reductions in their suitable climatic and land use and cover conditions were the primates *A. palliata* and *A. pigra*, the bat *B. io* and the marsupials *Ch. minimus* and *Ca. derbianus*. Primates *A. palliata* and *A. pigra* are already considered vulnerable and endangered by the IUCN, respectively. The bat *B. io* is considered to be vulnerable, and both species of marsupials are considered in the category of least concerned by the IUCN (2021) (Appendix S2). Our study identified these 20 species as the most threatened of the terrestrial mammals occurring in Mexico. We strongly encourage immediate and long‐term conservation actions for each of these species.

Overall, important reductions in areas holding projected suitable climatic and habitat conditions in species potential distributions pose an increasing threat for their long‐term conservation (Tables 2 and 3; Appendices S1 and S2). A species limited full‐dispersal assumption is more realistic than a non‐dispersal assumption, as evidence shows species follow their climatic niche (Antão et al., 2022). However, even under a non‐dispersal assumption, suitable climatic condition reductions only reached an additional 10% (Table 3; Appendix S8). We found higher reductions in species potential distributions projected under LUCC/WP than by CC; LUCC/WP assumes that rainfed agricultural and cattle grasslands are unsuitable habitats for terrestrial mammals to establish resident and reproductive populations (Linero et al., 2020; Mayani‐Parás et al., 2022; Peterson et al., 2011). This result contrasts with the study of Zamora Gutierrez et al. (2018) in which CC appeared to have a stronger negative effect than LUCC. We argue that CC and LUCC need to be addressed as potential additive impacts on biodiversity conservation (Ureta et al., 2012; Zamora Gutierrez et al., 2018). If future projected unsuitable climatic and habitat conditions increase, then there will be limited options for species and populations to persist, unless they adapt to new local conditions, disperse to areas with more favorable conditions, or go extinct. These alternatives will have a differential impact on species of terrestrial mammals, depending on their adaptive capabilities for new conditions and/or their dispersal abilities to shift their distributions to more favorable habitats (Peterson et al., 2001, 2011). Furthermore, future LUCC projects an increase in areas of rainfed agriculture and cattle grassland. The assumptions of these alternative scenarios (e.g., LUCC/WP and LUCC/WoP) will have a differential impact on species of terrestrial mammals. For example, large‐to‐medium‐sized mammals showed important reductions in projected suitable habitat conditions in their potential distributions, increasing future conservation threats (Figures 2 and 3; Table 4) (Hidalgo‐Mihart et al., 2019). Thus, if we assume that large‐to‐medium‐sized mammals establish permanent and reproductive populations in rainfed agriculture areas and cattle grassland (e.g., LUCC/WoP), then we will likely underestimate future species conservation threats. It can be more convenient for effective conservation planning to include a precautionary option, assuming that projected rainfed agriculture areas and cattle grassland are unsuitable habitats for maintaining resident and reproductive populations (e.g., LUCC/WP) (Linero et al., 2020; Mayani‐Parás et al., 2021). On the other hand, several small mammals frequently use rainfed agriculture areas for food search and sometimes establish populations as agricultural pests (Sánchez‐Cordero & Martínez‐Meyer, 2000). However, there is not enough biological information for most small mammals to assure that rainfed agriculture area will allow permanent and reproductive populations to become established (Hidalgo‐Mihart et al., 2019); for example, these populations may be exposed to adverse conditions, such as those created by the use of pesticides (Torquetti et al., 2021). LUCC that results in extensive area loss of suitable habitat for species currently represents the main cause of conservation threats for terrestrial mammals in Mexico (Chacón‐Prieto et al., 2021; Cuervo‐Robayo & Monroy‐Vilchis, 2012; Mayani‐Parás et al., 2021, 2022) and in other Neotropical biodiversity hotspots (Galetti et al., 2021; Linero et al., 2020).

High reductions in projected species‐rich areas under CC and LUCC scenarios will likely result in a cascading decay on ecosystem function and provision of environmental services, affecting socioeconomic activities and human well‐being (Bogoni et al., 2020; Supp et al., 2012). Similarly, cross‐time shifts in projected climatic and habitat suitability of species‐rich areas suggest future changes (gain and loss) in species compositions and turnover, reshaping species communities with unknown regional consequences in ecosystem functioning and provision of environmental services (Hillebrand et al., 2018). Efficient conservation efforts should not only focus on preserving a specific number of species, but also in the conservation of phylogenetic and functional diversity, which highlights the importance of incorporating different dimensions of biodiversity into conservation planning (Prieto‐Torres et al., 2021). We believe that our modeling exercise provides an information platform for identifying new projected priority areas for conservation (Hillebrand et al., 2018; Yu et al., 2021).

### 4.2 Risk in taxonomic group diversity

The taxonomic group diversity of orders of terrestrial mammals showed a differential impact due to CC. It is important to highlight that orders that include few species require special conservation priority; if one or two of the species showed a high risk, then the entire order is at risk. This is the case for the orders Cingulata (two species), Perissodactyla (one species), Primates (three species), and Pilosa (two species) that ranked in the top decile of highest risk. The order Cingulata includes the nine‐banded armadillo (*Dasypus novemcinctus*) listed as Least Concerned with stable populations, and the northern naked‐tailed armadillo (*Cabassous centralis*) listed as Data Deficient with decreasing populations. The order Perissodactyla includes the baird's tapir (*Tapirus bairdii*) listed as Endangered with decreasing populations. The order Primates includes the mantled howler monkey (*A. palliata*), the Guatemalan black howler (*A. pigra*), and the Geoffroy's spider monkey (*Ateles geoffroyi*), listed as vulnerable and endangered (the latter two species) with decreasing populations (IUCN, 2021) (Appendix S1). It is likely that most species belonging to these orders will face high conservation threats in the coming decades.

On the other hand, the taxonomic groups that include more species ranking in the top decile of highest risk were Eulipotyphla (34 species), Didelphimorphia (eight species), Artiodactyla (nine species), and Lagomorpha (11 species) (Table 4). The order Eulipotyphla is a speciose taxonomic group composed of shrews that show high habitat specialization and limited dispersal capabilities. These characteristics can make it difficult for species to disperse to future suitable climatic conditions due to limited population dispersal in response to habitat fragmentation (Guevara & Sánchez‐Cordero, 2018a). Moreover, many species of shrew (genus *Cryptotis*) are restricted to highly endangered habitats, such as montane cloud forest (Guevara & Sánchez‐Cordero, 2018a, 2018b). Thus, the current conservation status of several endemic species of shrews (Mayani‐Parás et al., 2022) can accelerate future conservation threats under CC and LUCC scenarios, as shown in this study.

The order Didelphimorphia includes species of marsupials ranging from species showing high ecological flexibility and wide distribution (e.g., *Didelphis virginiana*, *D. marsupialis*, and *Marmosa mexicana*) to highly ecologically specialized species showing restricted distributions (e.g., *Tlacuatzin canescens* and *Ca. derbianus*). Most species of Didelphimorphia are distributed in tropical forests (Sánchez‐Cordero et al., 2014), where current deforestation rates are increasing in Mexico (Fernández‐Montes de Oca et al., 2022). The current loss and fragmentation of habitat can significantly contribute to the increasing threat projected under future CC and LUCC scenarios. This suggest that tropical species of marsupials need urgent conservation attention, as described above for *Ca. derbianus*.

The order Artiodactyla includes large‐to‐medium‐sized terrestrial mammals with important ecological roles of consuming a large biomass of plants, seeds, and fruits in different ecosystems (Hester et al., 2006). Large‐sized herbivores, such as the bison *Bison bison* in grasslands, the white‐tailed deer *Odocoileus virginianus* in temperate and tropical forests, and the pronghorn *Antilocapra americana* in xeric shrublands (Lacher et al., 2019), are known to play a fundamental ecological role in diverse ecosystems by dispersing nutrients such as phosphorus, calcium, and sodium. Thus, population decreases of large herbivores due to future CC and LUCC scenarios are likely to increase the risk of energy flow and nutrient cycling reductions (Lacher et al., 2019). Furthermore, large‐sized species of herbivores are important prey for carnivores. Nearly half of the biomass of jaguar diets includes the ungulates *Mazama temama* and *Pecari tajacu*, while *P. tajacu* and *O. virginianus* contributed to half of the diet of pumas in southeast Mexico (Ávila‐Nájera et al., 2018). *M. temama* ranked at the top decile of highest risk under CC, and *O. virginianus* appeared in over 80% of the scenarios evaluated in the top decile at highest risk (Tables 2 and 3; Appendices S1 and S2). At least 50% of Artiodactyla species are at a national (SEMARNAT, 2010) or international (IUCN, 2021) threatened risk category. Thus, according to these projections, it is likely that more species of large‐to‐medium‐sized Artiodactyls will face a conservation threat in the coming decades with a corresponding negative impact on large‐sized top predators (Lacher et al., 2019).

The order Lagomorpha (rabbits, jackrabbits, and hares) includes 11 species ranging from endemics with highly restricted distributions (e.g., the volcano rabbit, *Romerolagus diazi*, and the Tehuantepec jackrabbit, *Lepus flavigularis*) to widely distributed species as *Sylvilagus floridanus*, *S. brasiliensis*, *L. californicus*, and *S. cunicularius* (Sánchez‐Cordero et al., 2014; Velázquez, 2012). Most species conform to large population abundances although illegal hunting and habitat loss are having a negative impact on several species of lagomorphs, such as *L. callotis*, *L. flavigularis*, and *R. diazi* (Mayani‐Parás et al., 2021; Velázquez, 2012), putting some species under a current high risk of extinction, such as the microendemic Omilteme cottontail rabbit *S. insonus* (Velázquez, 2012). Other orders of mammals are experiencing habitat loss and illegal hunting, which pose increasing challenges that should be incorporated in future conservation strategies (Chacón‐Prieto et al., 2021; Mayani‐Parás et al., 2021, 2022; Sánchez‐Cordero et al., 2014).

### 4.3 Risk in functional group diversity

Functional groups that include few species also need special conservation priority, such as the myrmecophagous (three species) and piscivores (seven species) (Table 3; Appendix S7); if only few species ranked high in risk, then the entire functional group is at risk. Specifically, myrmecophagous ranked in the top decile of highest risk and showed low redundancy (few species) and consequently less potential compensation of their role in ecosystems (Cumming & Child, 2009). Meanwhile, functional groups that include more than nine species ranking high in risk scores were insectivores under canopy, browsing herbivores, and frugivores–granivores (Table 4; Appendix S7). Species included in these functional groups play an important ecological role in predator–prey interactions, ecosystem functioning, and provision of environmental services. For example, frugivores–granivores and nectivorous mammals pollinate many wild (Ortega‐Baes & Godínez‐Alvarez, 2006; Saldaña‐Vázquez & Ortega‐García, 2021) and domesticated plant species of relevant social and economic values (Ha, 2014). Frugivore–granivores also play a crucial role in fruit and seed dispersal in many plant species occurring in different ecosystems (Lacher et al., 2019). If these scenarios of high risk for frugivore–granivores and nectivorous species of terrestrial mammals facing adverse climatic and habitat conditions are validated in the coming decades, important ecological, social, and economic consequences should be expected (Saldaña‐Vázquez & Ortega‐García, 2021; Trejo‐Salazar et al., 2016; Tremlett et al., 2021; Zamora Gutierrez et al., 2018). The ecological services provided by frugivore–granivores and pollinating nectivorous terrestrial mammals have been widely recognized by the Mexican government (SADER, 2021). There is a need to establish governmental policies addressing the pollination crisis that will likely be exacerbated in the coming decades (Neuschulz et al., 2016).

Insectivorous mammals play a fundamental role in controlling insect populations that can cause high socioeconomic costs due to crop loss. For example, some studies have estimated the high socioeconomic costs of population extirpations of insectivorous bats (Ramírez‐Bautista et al., 2020; Zamora Gutierrez et al., 2018). It has also been suggested that insectivorous terrestrial mammals help mitigate insect‐borne diseases by reducing oviposition (Lacher et al., 2019). Even though many insectivore species of bats might survive in a landscape transformed to agriculture, their exposure to pesticides can become an increasing threat (Torquetti et al., 2021). Rainfed agricultural areas are projected to increase in the coming decades (Mendoza‐Ponce et al., 2018) with a corresponding increase in the use of pesticides for pest control. This situation will affect insectivorous species of bats and shrews (through bio‐amplification), increasing their vulnerability to changes in their environment (Racero‐Casarrubia et al., 2021). If rainfed agricultural areas expand, then there is a strong need to move forward with the implementation of agroecological practices, including integrated pest management (Ha, 2014). Furthermore, herbivores play a fundamental role as a primary consumer of vegetation in many ecosystems, preserving a balance between vegetation communities and this group of terrestrial mammals (Lacher et al., 2019; Ripple & Beschta, 2012; Van Valkenburgh et al., 2016). Given that many species of large‐to‐medium‐sized herbivores (e.g., deer, hares, and rabbits) conform large populations, they consume large quantities of weeds, which prevent these invasive species from establishing resident populations and negatively affecting native plant species in many ecosystems (Lacher et al., 2019). Herbivores also include a large list of large‐to‐medium‐sized prey for many species of terrestrial vertebrate predators, such as mammalian carnivores, raptors, and snakes in complex predator–prey interactions in most ecosystems represented in Mexico (Lacher et al., 2019; Sánchez‐Cordero et al., 2014).

Large‐to‐medium‐sized species of terrestrial mammals were at higher risk under future CC and LUCC scenarios (Table 3), coinciding with observed trends reported in studies conducted under current climate and LUCC scenarios (Munguía et al., 2016). Species belonging to this body size category usually require large areas of suitable habitats used for territorial behavior and hunting needs (Ewer, 1998), posing high current and future conservation challenges in Mexico (Munguía et al., 2016). If species from this group increase their risk, resulting in decreasing local population abundance, we can expect important disruptions in predator–prey interactions in large areas of Mexico, with profound ecological implications (Erlinge et al., 1984; Estes, 1996; Ripple & Beschta, 2012). Further studies must also focus on species‐by‐species cases, where species belonging to several functional groups at risk deserve conservation attention. For example, the American beaver, *C. canadensis*, is a habitat‐specialized semi‐aquatic species with a highly restricted distribution in Mexico belonging to three functional groups at risk (herbivorous browser, semi‐aquatic, and large‐to‐medium‐sized body) (Appendices S2 and S4). Despite the fact that this species is considered to be least concerned (IUCN, 2021), its important functional and ecological role and highly restricted distribution in Mexico merit conservation priority.

Finally, our study provides an information platform for discussing conservation strategies involving governmental agencies, NGOs, academia, stakeholders, landowners, and the general public to address the projected impacts of future CC and LUCC scenarios on terrestrial mammals at the regional and national levels. Several countries have highlighted the importance of incorporating sound sustainable programs with the coexistence of wildlife to ensure long‐term biodiversity conservation (Carter & Linnell, 2016).

### 4.4 Limitations of the study

We are aware of the uncertainty associated with GCMs, and that is one reason why we chose two contrasting CC circulation models: the CAN, which proved to adequately simulate the observed climate for Mexico and the BCC (Altamirano del Carmen et al., 2021; Shepherd et al., 2018). Nonetheless, we acknowledge that our modeling extrapolation and results should be interpreted with caution due to the uncertainty associated with the GCMs (Appendix S1). We are also aware of the fact that it would have been ideal to use the same scenarios (SSPs and RCPs) in the distribution modeling and LUCC. However, the LUCC scenarios for Mexico were built before CMIP6 was released (Mendoza‐Ponce et al., 2018), and models were conducted with GCMs that had not been evaluated as robust in the Northern Hemisphere. In this study, we used the GCM with highest similarity to CAN, which is a previous generation of the GCM used to conduct our analyses (HAD). Climate does not appear to play a crucial role in LUCC scenarios (Mendoza‐Ponce et al., 2018), and the layers used have average trends with no additional assumptions, and thus, we believe that the models can be combined without losing consistency. Furthermore, ecological niche modeling contains another source of uncertainty in the threshold selected to build binary maps, which has an important effect on the area of suitable habitat available for species in current and future climatic conditions. However, even though that threshold selection has been recognized as a source of uncertainty in CC assessments, it has been shown not to be the most influential (Thuiller et al., 2019).

Our study included the terrestrial mammals occurring in Mexico, of which an important proportion of species are non‐endemic. Despite the fact that the correlative approach used included the entire American continent, we restricted our analyses to the species potential distributions occurring in Mexico. This is important to establish because species identified as having high or low projected risk nationwide can show different risk values elsewhere in their distributions. Our risk index also assumed that the species included showed niche conservatism and full dispersal inside their corresponding geographic calibration and transference area (Peterson et al., 2011). We referred to this as a limited full‐dispersal assumption, which indirectly incorporated species dispersal abilities into our modeling exercise. However, we also incorporated the ecological niche modeling under a non‐dispersal assumption to include scenarios of climatic suitability of species unable to disperse. Our risk index also excluded species losing their entire suitable climatic condition under any combination evaluated, and located these species with highest exposure. Moreover, it is quite challenging to determine weighting factors quantifying the importance of each input variable. Rather, those weighting factors should most probably be different for each species. We are aware that building risk scores and indexes of species, taxonomic, and functional group diversities, and identifying shifts of species‐rich areas under CC and LUCC scenarios are merely projections. Our projections in species risk are more likely to better apply for short‐term (e.g., 2030) than long‐term (e.g., 2070) cross‐time CC and LUCC scenarios.

## CONFLICT OF INTEREST

The authors declare no competing interests.

## Supporting information


Appendix S1

Appendix S2

Appendix S3

Appendix S4

Appendix S5

Appendix S6

Appendix S7

Appendix S8

## Data Availability

All data used for the analyses in this paper will be available through Supplementary Information and GitHub: https://github.com/Edgarandre5/R_geoanalysis. All of our Supplementary Information is available in the following link: https://zenodo.org/record/7027856. Maps generated are available at https://www.conabio.gob.mx/informacion/gis/. In the "Acervo" navigate through: Biodiversidad, Cambio global, Cambio climático, Especies, Mamíferos.
