# Supplementary material for: Species, taxonomic, and functional group diversities of terrestrial mammals at risk under climate change and land‐use/cover change scenarios in Mexico
Source: Glob Chang Biol. 2022 Sep 13;28(23):6992–7008. doi: 10.1111/gcb.16411 (PMC9826092; doi:10.1111/gcb.16411)
Supplement: Supplementary file 2 — Appendix S2 [file GCB-28-6992-s005.docx]

Species, taxonomic, and functional group diversities of terrestrial mammals at risk under climate change and land use/cover change scenarios in Mexico

Authors: Carolina Ureta^1,2,*,†^, Mercedes Ramírez-Barrón^3,*^, Edgar Andrés Sánchez-García^3^*, Ángela Cuervo-Robayo^4^, Mariana Munguía^4^,  Alma Mendoza^1^, Carlos Gay^1^, Víctor Sánchez-Cordero^3, †^

**Supplementary Material 2**

- This file contains:
  - 6 supplementary figures and 3 tables.
  - 6 supplementary excel tables (Supplementary material S2, Supplementary material S3, Supplementary material S4 and Supplementary material S5).
  - 4 R codes available at GitHub with the identifier https://github.com/Edgarandre5/R_geoanalysis

**Table 1.** Changes in temperature and precipitation for 2030, 2050, and 2070 between present-day and future climate change scenarios evaluated, respectively. T°C: mean annual temperature, ΔT°C: difference between present day scenario and the scenario evaluated, P: annual precipitation, and Δ P: difference between annual precipitation from a present-day scenario and the scenario evaluated.

**Table 2.** Losses in species rich areas (> 75 species) between present-day scenario and all possible future climate change scenarios evaluated. Scenario: all future scenarios evaluated, Time: time horizons evaluated and % Reductions: percentage of potential rich areas expected to be reduced in the scenario evaluated.

**Table 3.** Species rich areas (>75 species) under unsuitable habitat for species due to land use and cover change (HADGEM2). Climate change scenario (CC): all scenarios evaluated (SSP2 4.5 BCC-CSM2.MR/ CanESM5; SSP5 8.5 BCC-CSM2.MR/ CanESM5), Year: years evaluated 2030 (2021–2040), 2050 (2041–2060), and 2070 (2081–2100). Land-use and cover changes (LUCC) including with precautionary option (LUCC/WP), and without precautionary option (LUCC/WoP). Inside LUCC/WP or LUCC/WoP = inside unsuitable habitat for species due to land use change area LUCC/WP, and LUCC/WoP. Total: total number of pixels with potential species rich area (>75 species). See Methods for details.

| **CC scenario** | **Year** | **LUCC/WP-LUCC/WoP** | **Inside LUCC/WP-LUCC/WoP** | **Total** | % |
| --- | --- | --- | --- | --- | --- |
| 245BCC | 2030 | WP | 15330 | 30450 | 0.503 |
| 245CAN | 2030 | WP | 16619 | 33233 | 0.500 |
| 585BCC | 2030 | WP | 15576 | 31423 | 0.496 |
| 585CAN | 2030 | WP | 16447 | 32825 | 0.501 |
|  |  | WP |  |  |  |
| 45BCC | 2050 | WP | 14490 | 26931 | 0.538 |
| 245CAN | 2050 | WP | 17056 | 31560 | 0.540 |
| 585BCC | 2050 | WP | 13049 | 24437 | 0.534 |
| 585CAN | 2050 | WP | 14512 | 27905 | 0.520 |
|  |  | WP |  |  |  |
| 245BCC | 2070 | WP | 16384 | 29119 | 0.563 |
| 245CAN | 2070 | WP | 16595 | 29088 | 0.571 |
| 585BCC | 2070 | WP | 13129 | 23243 | 0.565 |
| 585CAN | 2070 | WP | 12554 | 22072 | 0.569 |
|  |  |  |  |  |  |
| 245BCC | 2030 | WoP | 3107 | 30450 | 0.102 |
| 245CAN | 2030 | WoP | 3298 | 33233 | 0.099 |
| 585BCC | 2030 | WoP | 3182 | 31423 | 0.101 |
| 585CAN | 2030 | WoP | 3236 | 32825 | 0.099 |
|  |  | WoP |  |  |  |
| 45BCC | 2050 | WoP | 2946 | 26931 | 0.109 |
| 245CAN | 2050 | WoP | 3531 | 31560 | 0.112 |
| 585BCC | 2050 | WoP | 2607 | 24437 | 0.107 |
| 585CAN | 2050 | WoP | 2788 | 27905 | 0.100 |
|  |  | WoP |  |  |  |
| 245BCC | 2070 | WoP | 3244 | 29119 | 0.111 |
| 245CAN | 2070 | WoP | 3366 | 29088 | 0.116 |
| 585BCC | 2070 | WoP | 2749 | 23243 | 0.118 |
| 585CAN | 2070 | WoP | 2319 | 22072 | 0.105 |

**Figure 1.** Steps to develop land-use and cover change scenarios. LUCC: Land-use and cover change scenarios.


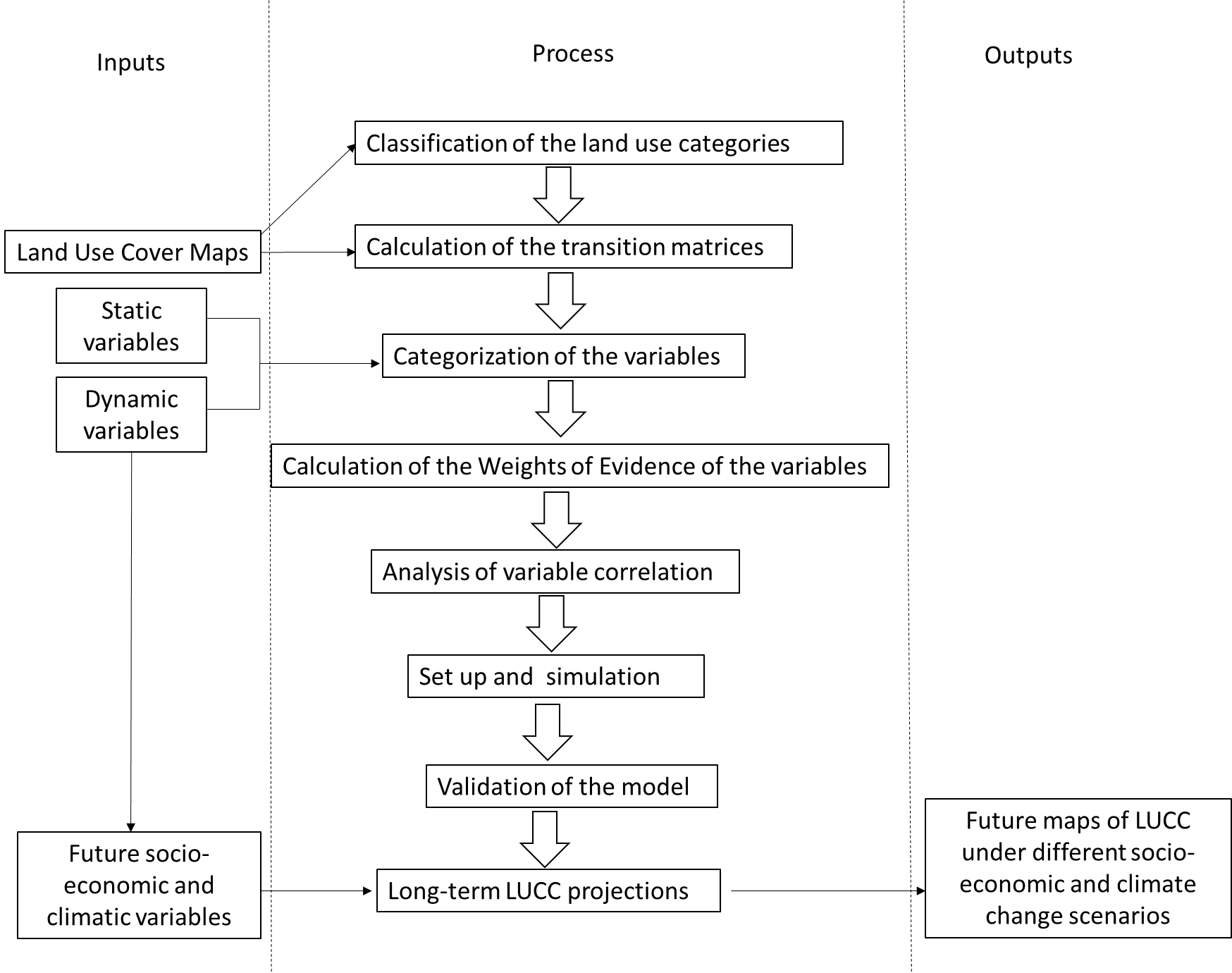


**Figure 1.** Species rich-areas projected for 2030 (2021–2040), 2050 (2041–2060), 2070 (2081–2100). A-D: Species-rich areas with suitable climatic conditions, E-H: Species-rich areas with suitable climatic conditions and suitable habitat conditions, assuming agriculture areas unsuitable habitat (LUCC/WP) for species. A, C, E, G: BCC-CSM2.MR; B, D, F, H: CanESM5.

**
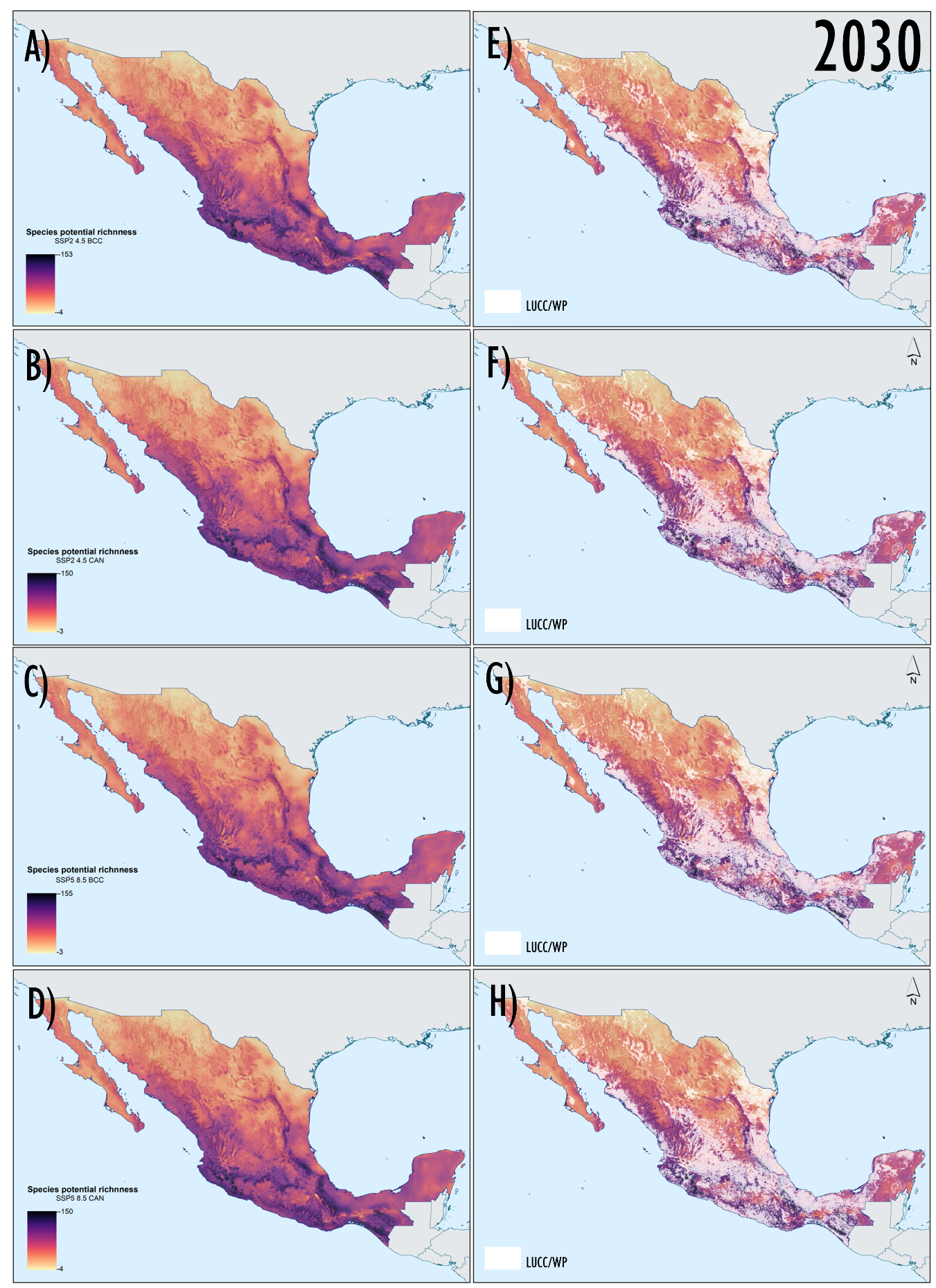
**


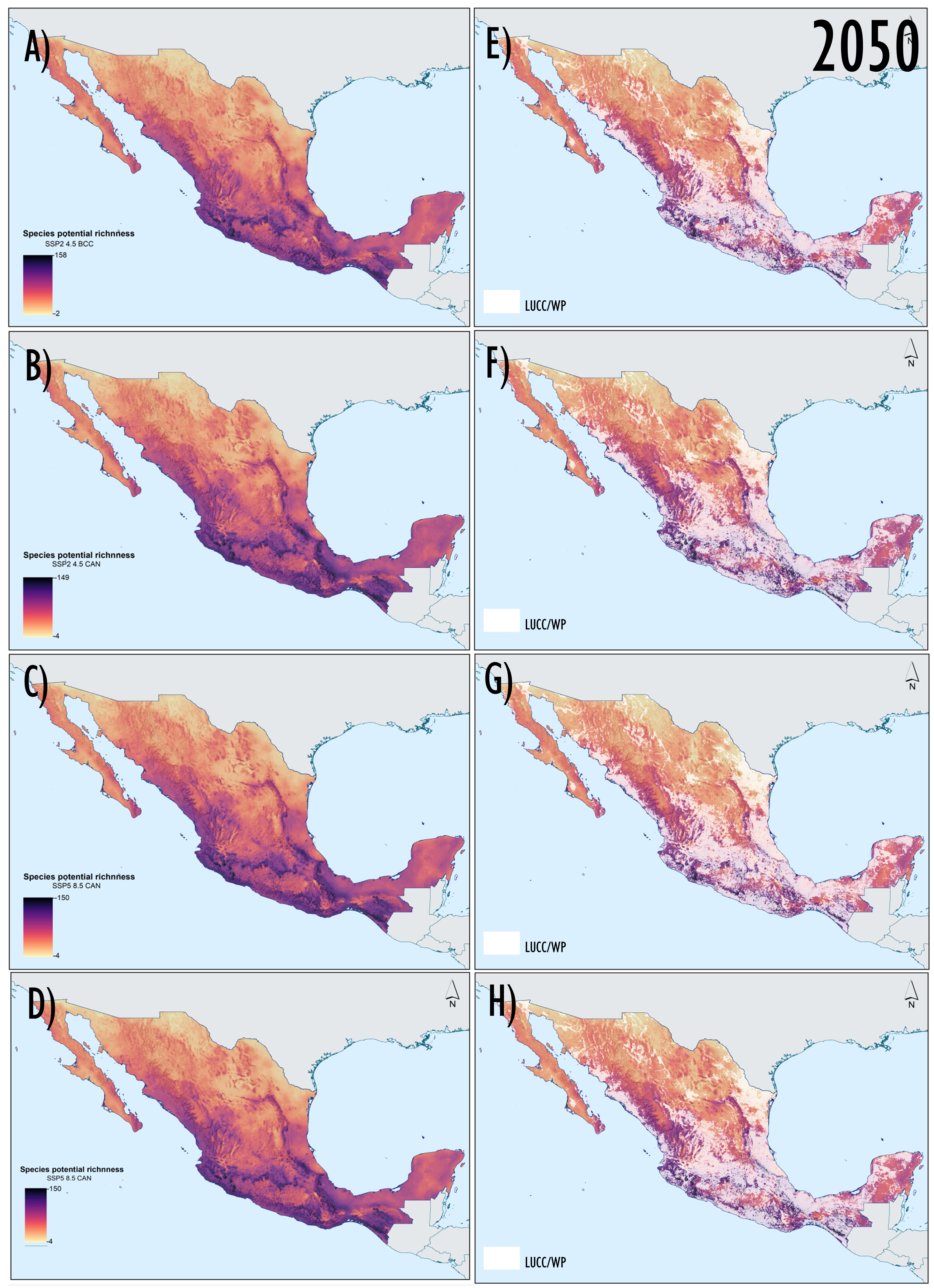

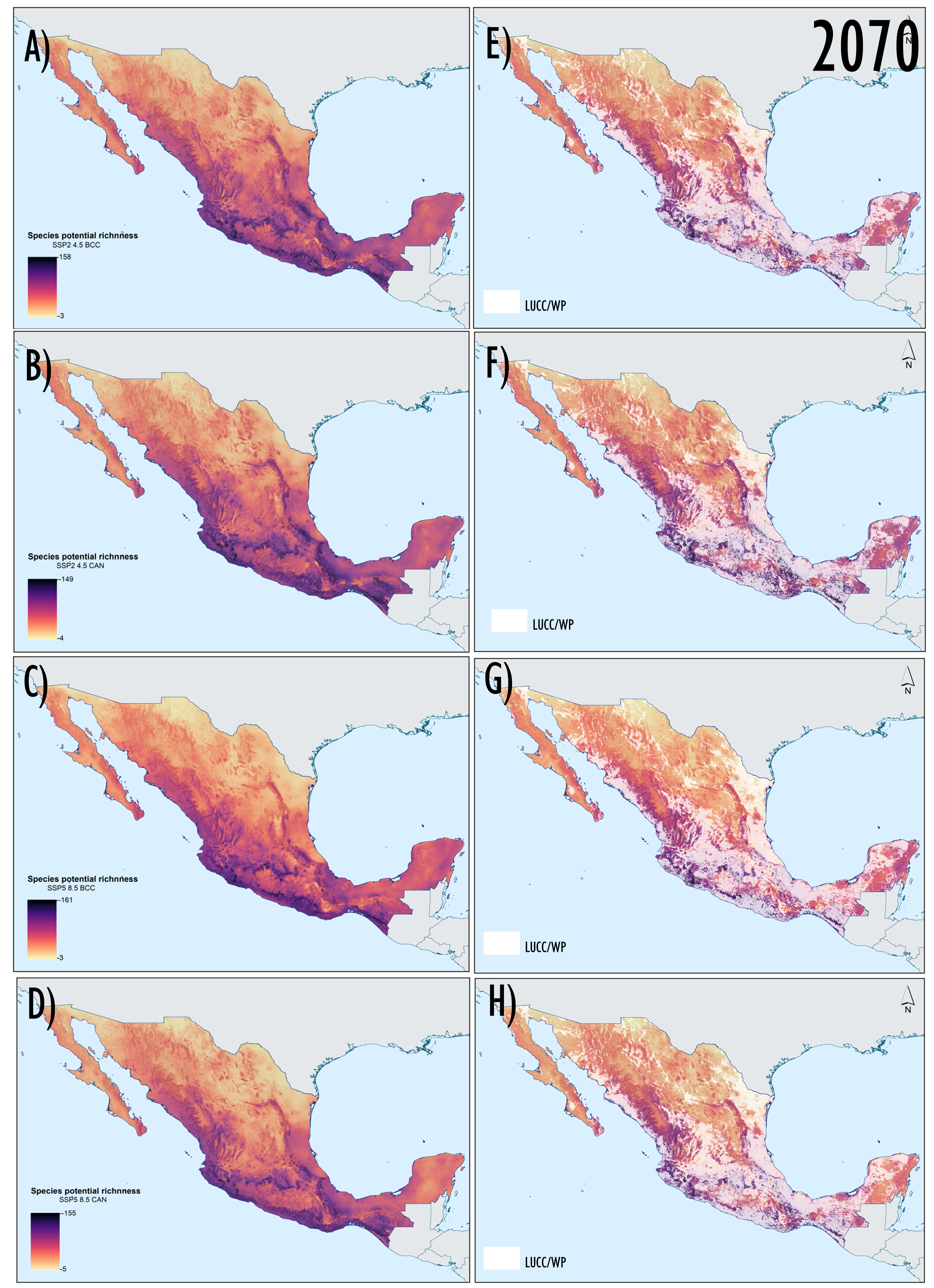


**Figure 2.** Geographic areas with higher losses in potential distribution areas (PDA) under different climate change scenarios and three-time projections: 1) 2030 (2021–2040), 2) 2050 (2041–2060) and 3) 2070 (2081–100). Land-use and cover changes, assuming temporal agriculture and livestock areas unsuitable habitat for species (LUCC/WP). A-B: BCC-CSM2.MR; C-D: CanESM5. See Methods for details.


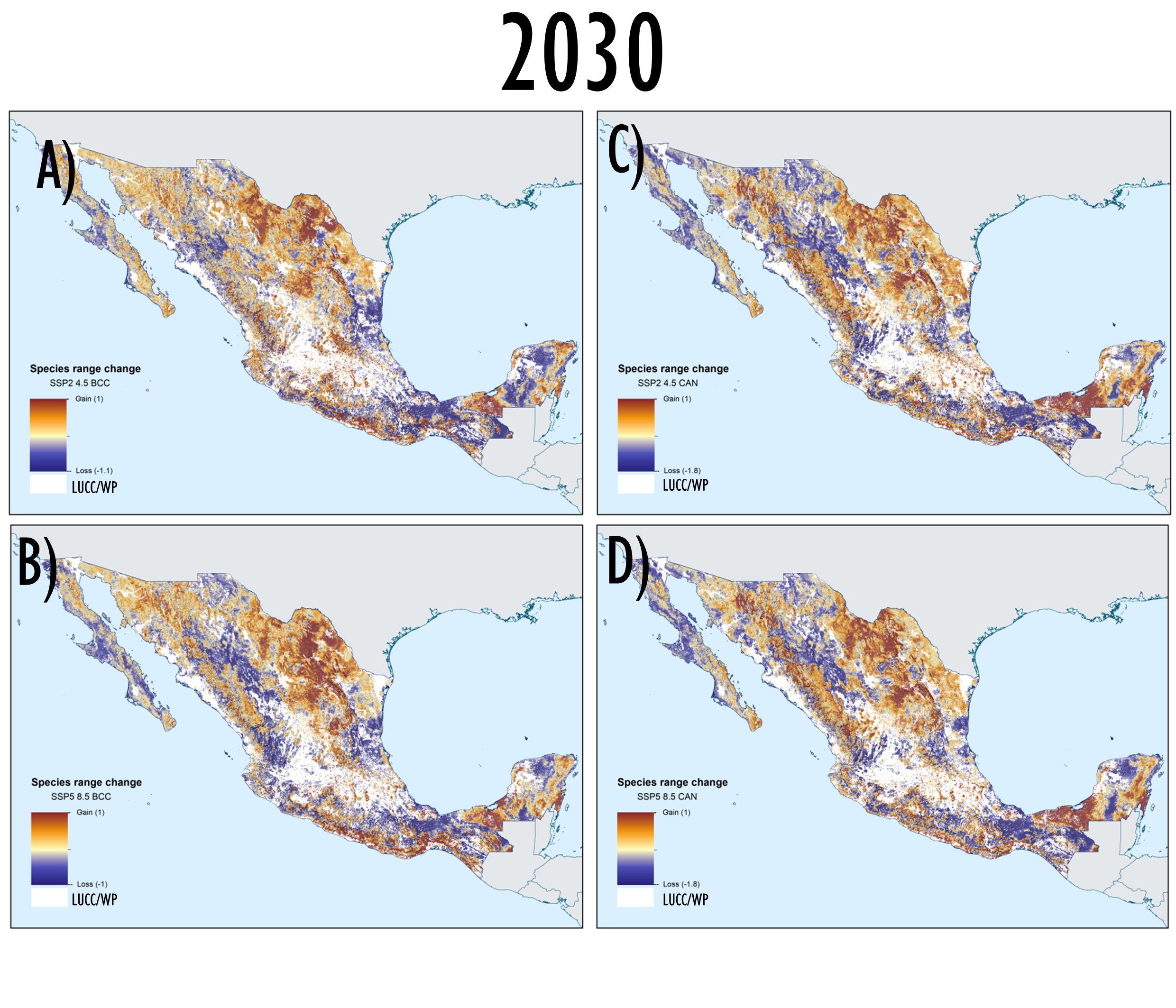

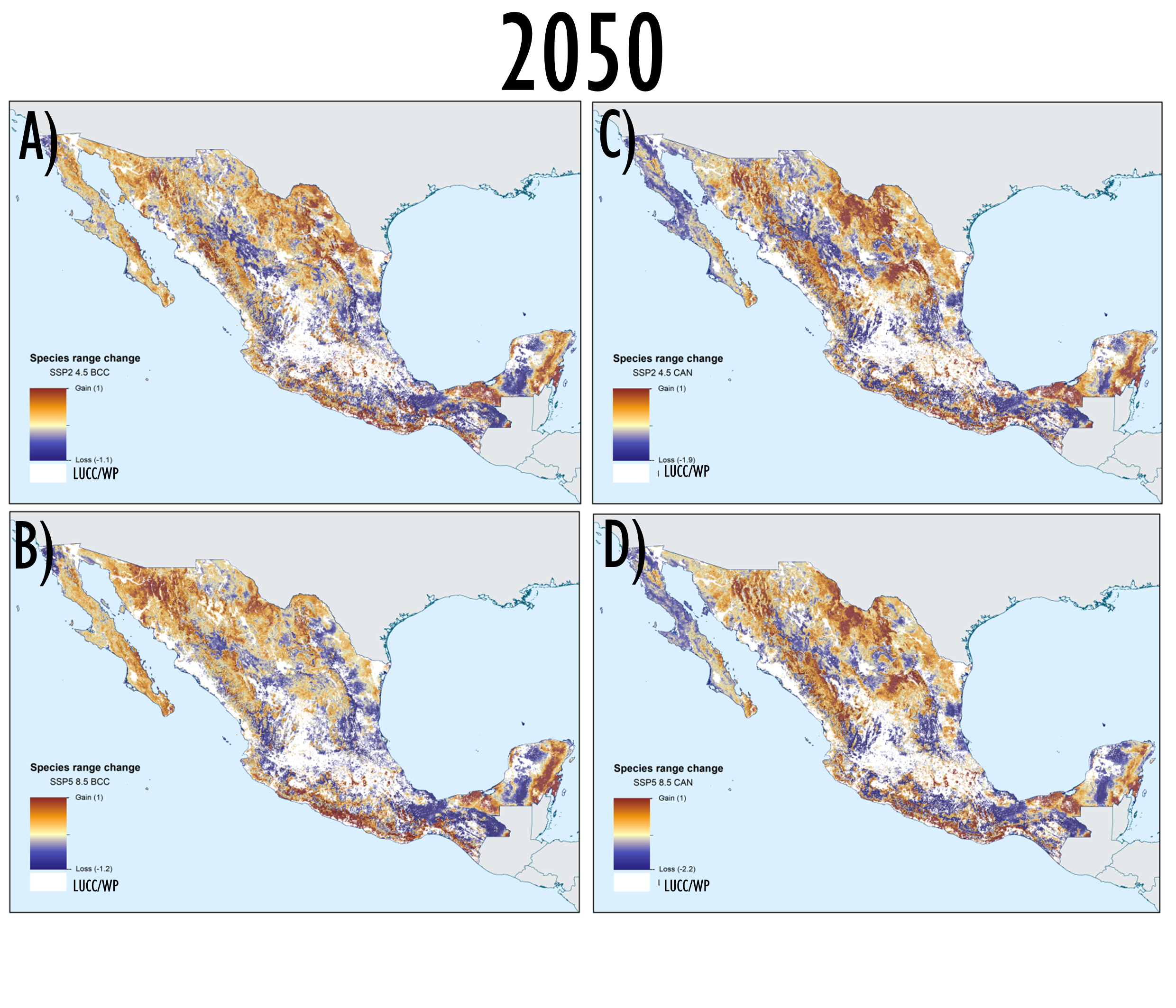

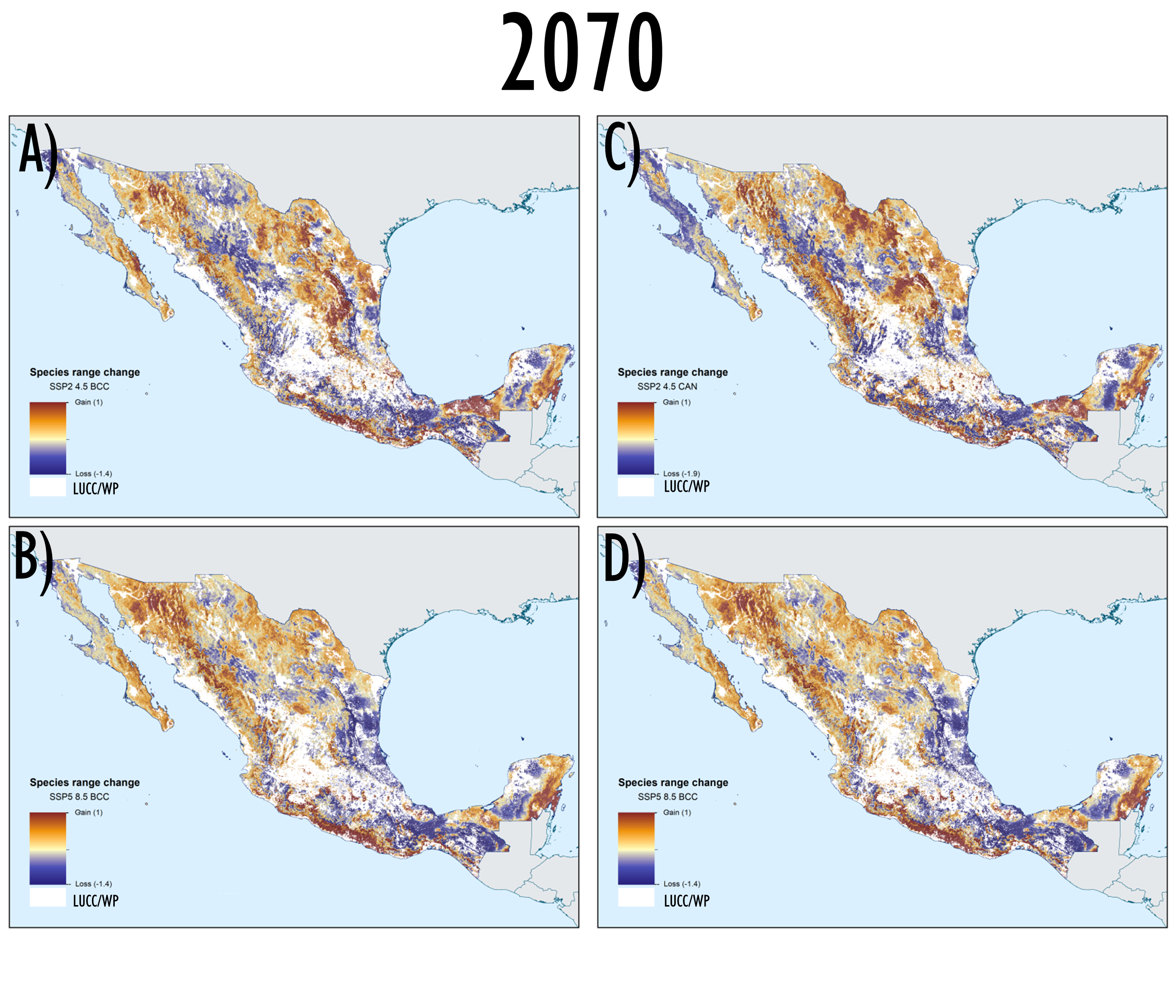


**Figure 3.** Differences between General Circulation Models simulations for (A) 2030 (2021–2040), (B) 2050 (2041–2060), and (C) 2070 (2081–2100) climate change scenarios in Mexico.


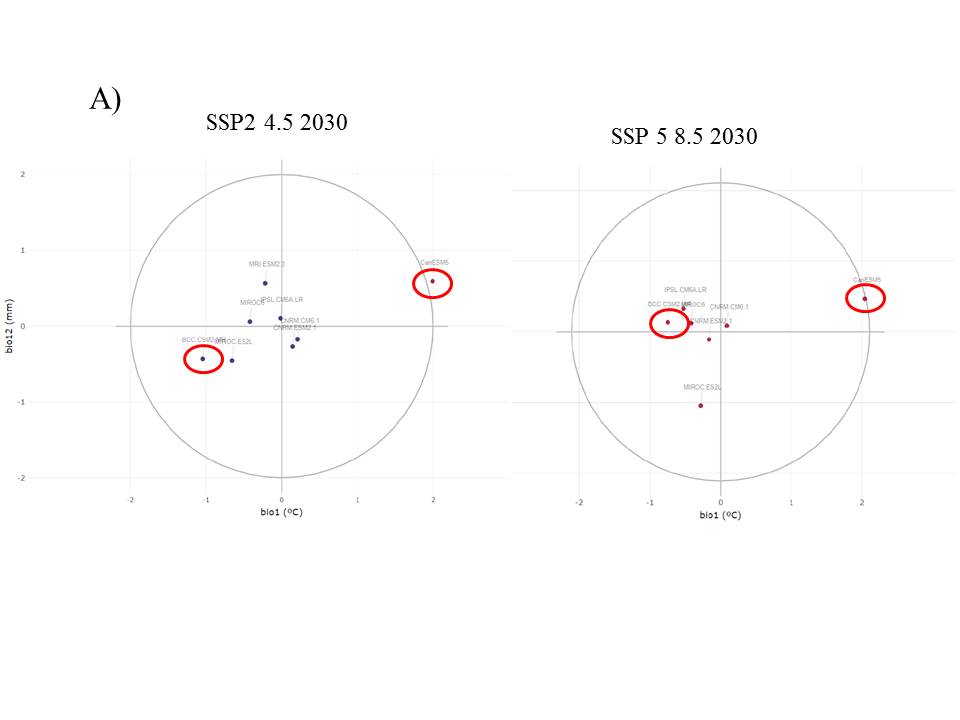


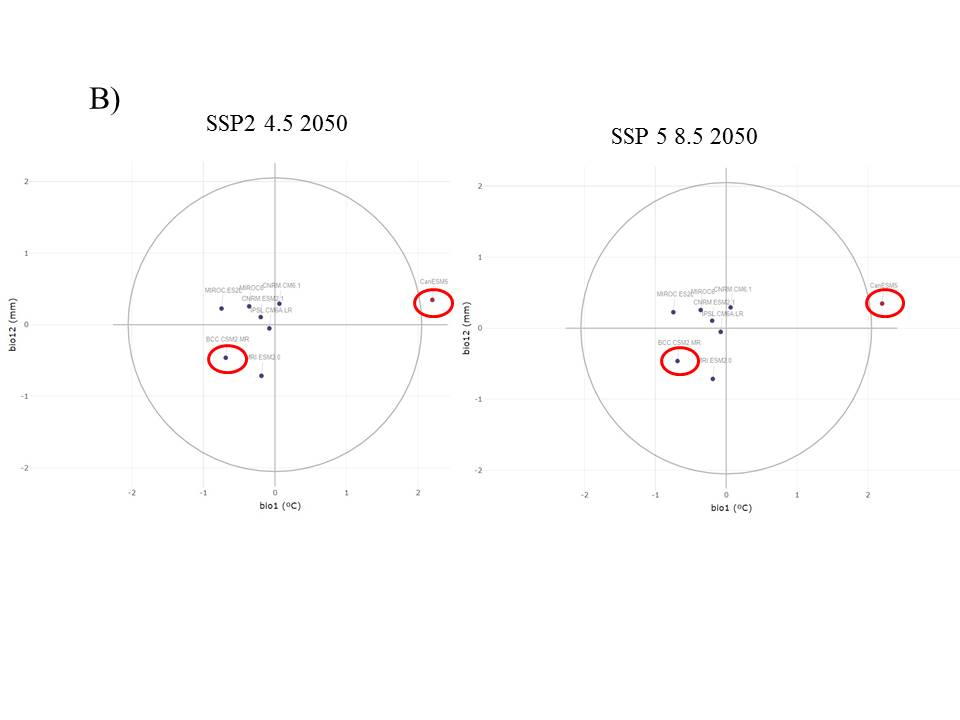


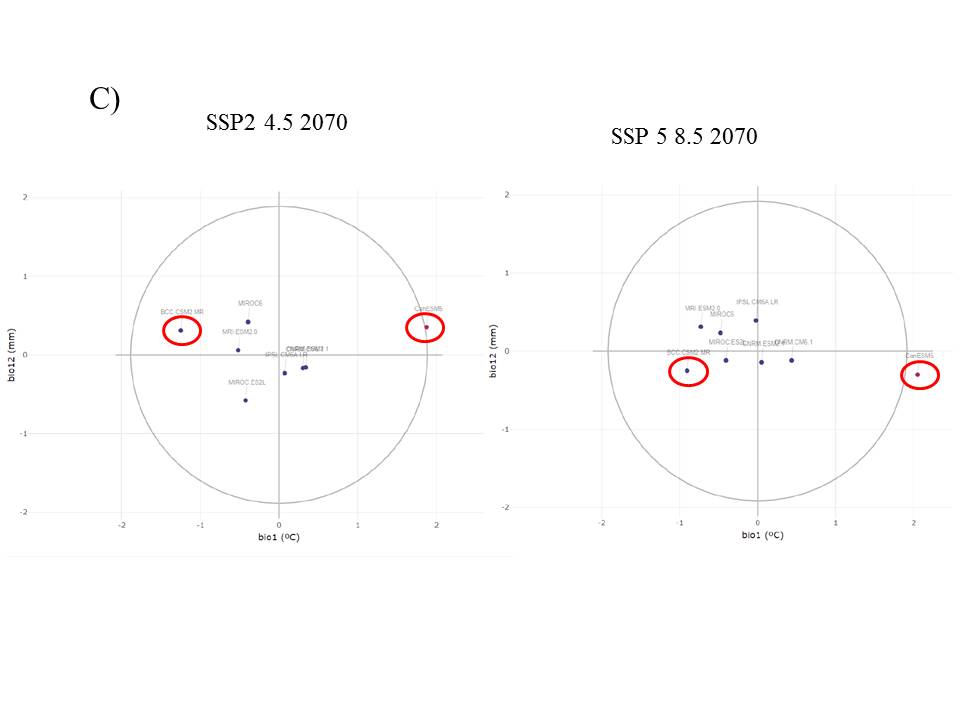


**Figure 4.** Statistical differences between risk scores obtained under BCC-CSM2.MR and CanESM5. Differences were carried out for all times (2030, 2050 and 2070) and scenarios (SSP245 and SSP585). To obtain the figure we used the function ggwithinstats of the package ggstatsplot (Patil, 2021).

| **A.** |
| --- |
| **** |
| **B.** |
| **** |
| **C.** |
| **** |
|  |

| **D.** |
| --- |
| **** |
| **E.** |
| **** |
| **F.** |
| **** |
|  |

**Figure 5. Identification of geographic areas with extrapolation probability.** The **red color** in figures: (a) represent that at least one variable is outside the range of the reference data, and (b) it is possible to visualize to which variables that area corresponds. For General Circulation Model BCC-CCM2-MR, we only present one figure because in all scenarios and times, the only variable that is outside its reference range is Bio2, and the geographic area outside the range is very similar across all combinations evaluated.

**Figure 6. General circulation models (GCMs), years and Shared Socioeconomic Pathways (SSPs).** 2030: 2021–2040, 2050: 2041–2060 and 2070: 2081–2100.

General circulation models

BCC-CSM2

CanESM5

2070

2030

SSP2 4.5

2050

2070

2030

2050

SSP5 8.5

SSP2 4.5

SSP5 8.5

SSP2 4.5

SSP5 8.5

SSP2 4.5

SSP5 8.5

SSP5 8.5

SSP2 4.5

SSP5 8.5

Patil, I. (2021). Visualizations with statistical details: The “ggstatsplt” approach. Journal of Open Source Software, 6(61), 3167.
